# Vaccinia-Virus-Based Vaccines Are Expected to Elicit Highly Cross-Reactive Immunity to the 2022 Monkeypox Virus

**DOI:** 10.3390/v14091960

**Published:** 2022-09-03

**Authors:** Syed Faraz Ahmed, Muhammad Saqib Sohail, Ahmed Abdul Quadeer, Matthew R. McKay

**Affiliations:** 1 Department of Electrical and Electronic Engineering, University of Melbourne, Parkville, VIC 3010, Australia; 2 Department of Electronic and Computer Engineering, The Hong Kong University of Science and Technology, Hong Kong SAR, China; 3 Department of Microbiology and Immunology, The Peter Doherty Institute for Infection and Immunity, University of Melbourne, Melbourne, VIC 3000, Australia

**Keywords:** monkeypox, vaccinia virus, vaccines, immunity, genetic similarity, T cells, neutralizing antibodies, epitopes, Dryvax, ACAM2000, MVA-BN

## Abstract

Beginning in May 2022, a novel cluster of monkeypox virus infections was detected in humans. This virus has spread rapidly to non-endemic countries, sparking global concern. Specific vaccines based on the vaccinia virus (VACV) have demonstrated high efficacy against monkeypox viruses in the past and are considered an important outbreak control measure. Viruses observed in the current outbreak carry distinct genetic variations that have the potential to affect vaccine-induced immune recognition. Here, by investigating genetic variation with respect to orthologous immunogenic vaccinia-virus proteins, we report data that anticipates immune responses induced by VACV-based vaccines, including the currently available MVA-BN and ACAM2000 vaccines, to remain highly cross-reactive against the newly observed monkeypox viruses.

## 1. Introduction

The monkeypox virus outbreak observed in 2022 (MPXV-2022) is highly distinctive. Unlike previous outbreaks of monkeypox, which were localized [1,2,3] and resulted in small numbers of infections due to limited ability for human-to-human transmission [4,5,6], the emerging outbreak has already resulted in over 52,000 confirmed cases, spanning more than 90 countries [7], in a few months since the first case was reported on 7 May 2022 [8]. On 23 July 2022, the World Health Organization (WHO) declared the 2022 monkeypox outbreak a global health emergency of international concern [9]. The underlying determinants of the MPXV-2022 outbreak remain unclear [10]; however, phylogenetic analyses [11] of genomic sequences reported on GISAID [12] from at least 15 countries place these samples in the West African clade of MPXV (MPXV-WA). This is surprising, given the historically low outbreak-causing potential observed for this particular clade [13,14,15].

Vaccines based on vaccinia virus (VACV), originally developed against smallpox, are one of the interventions available for preventing and controlling monkeypox. There are three major types of VACV-based vaccines. First-generation vaccines comprise live VACV, e.g., Dryvax, that were used for the eradication of smallpox in the last century [16]. While the WHO and several countries maintain a stockpile of these vaccines, their use against MPXV is not recommended due to safety concerns [17]. ACAM2000, a second-generation vaccine, is also based on live VACV, but has a better safety profile than the first-generation vaccines [18]. This vaccine is currently available in the US for use against monkeypox under an expanded access investigational new drug application [19]. A third-generation vaccine, Bavarian Nordic’s modified vaccinia virus Ankara (MVA-BN), is currently recommended by WHO and the US Centers for Disease Control and Prevention (CDC) in high risk groups for controlling the current monkeypox outbreak [17,19]. MVA-BN is a highly attenuated VACV-based vaccine incapable of replication in humans [20]. While safe and easier to administer than earlier generation vaccines, MVA-BN is currently available only in limited quantity [17].

Despite differences in replication and safety profiles, each of these VACV-based vaccines have been reported to induce high neutralizing antibody titers and strong T-cell responses among vaccinated individuals [18,20,21,22,23,24,25]. There is also some evidence from efficacy studies in humans, as well as in animal models, that these vaccines induce cross-reactive and protective immune responses against MPXV. In humans, the first-generation VACV-based vaccines were reported to offer 85% protection against MPXV during monkeypox outbreaks in Africa in the 1980s [4,26], while both the ACAM2000 and MVA-BN vaccines were shown to be protective against MPXV in animal models [27,28]. However, importantly, these efficacy results were reported against the Congo Basin clade of MPXV (MPXV-CB). For all VACV-based vaccines, there is a lack of scientific data on their cross-reactive immunity and efficacy, in both humans and animals, against viruses belonging to the MPXV-WA clade, which appears to be most relevant to the sequences observed in the current outbreak [11].

In this study, we aimed to investigate the expected cross-reactive immunity of VACV-based vaccines against the MPXV-2022 outbreak viruses. We used available genomic sequence and immunological data to quantify genetic similarities and differences between the VACV immunogenic proteins and their orthologs in the MPXV-2022 isolates. Given that VACV-based vaccines are known to elicit both antibody (humoral) and T-cell (cellular) responses in humans [18,21,22,23,24,25,29,30,31], we analyzed VACV proteins and epitopes known to be specific targets of antibodies and T cells.

While identifying a small number of mutations of potential immunological consequence, our data broadly indicates that MPXV-2022 is highly genetically conserved within immunogenic protein regions and epitopes of VACV. As such, the MPXV-2022 outbreak viruses will likely exhibit similar cross-reactive humoral and cellular immunity profiles upon vaccination with VACV-based vaccines to those observed against MPXV-CB upon vaccination with the first-generation vaccines. Based on this, the efficacy of these vaccines against MPXV-2022 might also be expected to be high.

## 2. Materials and Methods

### 2.1. Acquisition and Pre-Processing of Sequence Data

A total of 513 complete genome sequences of MPXV-2022 were downloaded from the GISAID database (https://www.gisaid.org/; accessed on 24 July 2022) (Table S1). The complete genome reference sequences for the VACV and MPXV-CB were downloaded from NCBI using the GenBank accession IDs NC_006998 and NC_003310 (Zaire-96-I-16), respectively. Similarly, the reference sequences for the vaccines ACAM2000 and MVA-BN were downloaded using GenBank accession IDs AY313847 and DQ983238, respectively. For the Dryvax vaccine, 11 sequences were downloaded from NCBI, with accession IDs JN654976 through JN654986, and their consensus sequence was used in the analysis. Similar to our previous works [32,33], an in-house bioinformatics pipeline was developed to align these nucleotide sequences and then translate them into amino acid residues according to the coding sequence positions provided along the reference sequence for VACV and the location of the gene (whether on the forward or reverse strand). MAFFT software was used to perform all multiple sequence alignments [34]. A total of 182 genes, with coding sequences, are described along the VACV reference sequence, and the corresponding translated genomic regions of MPXV-2022 sequences represent the 182 MPXV-2022 ortholog proteins.

### 2.2. Computing Genetic Similarity

Genetic similarities between any pair of nucleotide or protein sequences were computed from their pairwise sequence alignments. All positions within the pairwise alignment that had a gap were counted as insertion/deletion (indel). For nucleotide pairwise alignments, all positions where there was a mismatch of nucleotides were counted as single nucleotide polymorphisms (SNPs), while for protein pairwise alignments, all positions where there was a mismatch of residues were counted as substitutions. Genetic similarity was defined as the fraction of positions in the pairwise alignments that had no SNPs/substitutions or indels.

### 2.3. Acquisition of Epitope Data

VACV-derived B cell and T cell epitopes were searched on the Immune Epitope Database (IEDB) (https://www.iedb.org/; accessed 10 June 2022) [35] by querying for the virus species name: “Vaccinia virus” (taxonomy ID: 10245) from “human” hosts. The search was limited to include only experimentally determined epitopes [36] that were associated with at least one positive assay: (i) positive B cell assays for B cell epitopes, and (ii) positive T cell assays for T cell epitopes. For the B cell epitopes, only two epitopes were found (IEDB IDs: 735903 and 735904), both within the D8L protein. A literature search identified one additional VACV antibody epitope within D8L [37] reported to be targeted in humans. For the T cell epitopes, the search was restricted to epitopes with lengths between 9 and 21 residues, which covers the typical range of both HLA class I and class II epitopes. This search returned 388 T cell epitopes in total.

### 2.4. Selection of Immunogenic Proteins

The eight VACV proteins identified as targets of neutralizing antibodies in humans (A17L, A27L, A28L, A33R, B5R, D8L, L1R, and H3) have been widely reported in the literature [38,39]. The 121 VACV proteins identified as targets of T cells were obtained by mapping the 388 VACV-derived T cell epitope sequences onto the VACV protein sequences.

### 2.5. Visualization of Protein Crystal Structures

VACV protein crystal structures were obtained from the Protein Databank (www.rcsb.org; accessed on 24 July 2022), and the structural figures were made using PyMOL software (www.pymol.org; accessed on 22 June 2022).

## 3. Results

For assessing cross-reactive vaccine-induced immune responses against MPXV, we used the VACV reference sequence as a representative of VACV-based vaccines. This is motivated by the fact that VACV-based vaccines are known to elicit comparable immune responses [18,20,21,22,23,24,25], and almost all immunological data available in the literature is for VACV. Moreover, the VACV reference sequence exhibits ~98% genetic similarity (discounting indels) to the VACV-based vaccine sequences (Dryvax, ACAM2000, and MVA-BN) (Figure S1).

The MPXV-2022 sequences demonstrate a mean genetic similarity of ~84% to the VACV reference sequence (GenBank: NC_006998.1). The sequences contain ~3% SNPs and ~13% indels, which translates to ~6.5 k SNPs and ~27.5 k indels, due to the large genome sizes of MPXV-2022 and VACV (~200 kbp). Investigation of the genetic differences within immunogenic proteins that are targets of either B cells/antibodies or T cells can offer insights into the anticipated effects of this genetic variation on immune recognition by VACV-based vaccine-induced responses.

### 3.1. VACV Proteins Targeted by Neutralizing Antibodies Share High Sequence Similarity with MPXV-2022 Orthologs

Eight VACV immunogenic proteins are known to elicit neutralizing antibodies (NAbs) [38,39]. A subset of these has been used as an antigen in subunit vaccines against smallpox and monkeypox [40,41,42,43,44,45]. The similarity of the eight proteins, with respect to their orthologs in MPXV-2022 sequences and the MPXV-CB reference sequence, was evaluated. The latter serves as a meaningful reference, since the efficacy of the first-generation VACV-based vaccine against monkeypox outbreaks caused by MPXV-CB has been reported previously [4,26]. For all eight immunogenic proteins, there was a high genetic similarity (range: ~94% to ~98%) between VACV and both the MPXV-2022 consensus sequence and the MPXV-CB reference sequence (Table 1). The high genetic similarity observed for the MPXV-2022 consensus sequence was also observed when considering the complete set of available MPXV-2022 sequences (Figure S2).

**Figure 1:**
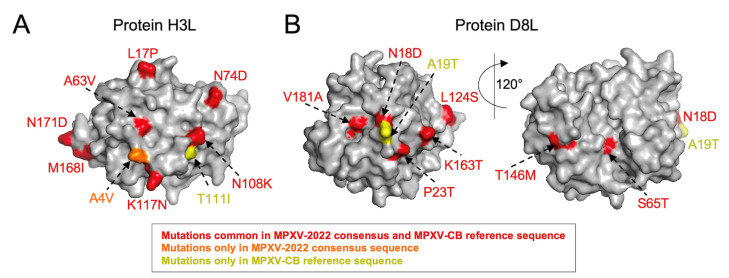
Mapping mutations observed in MPXV-2022 and MPXV-CB on the structure available for VACV (**A**) H3L [PDB ID: 5EJ0] and (**B**) D8L [PDB ID: 4E9O] surface proteins. The core structure of each protein is shown in gray, while mutations and their labels are colored according to the scheme in the legend.

**Figure 2:**
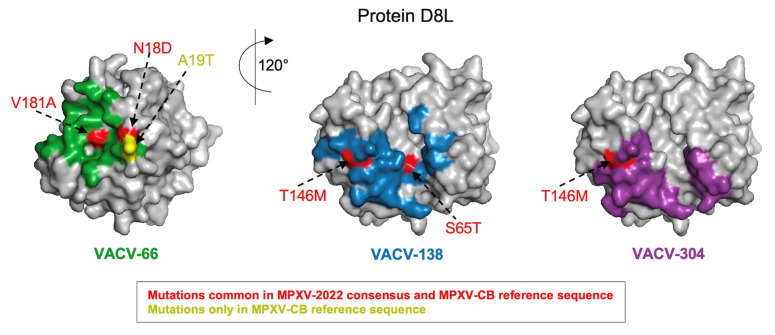
MPXV-2022 does not comprise any new mutations relative to the MPXV-CB reference sequence in the epitopes of the known D8L-specific antibodies. Mutations observed in MPXV-2022 and MPXV-CB are mapped on the epitope of the three known neutralizing antibodies (VACV-66, VACV-138, and VACV-304) in the D8L protein [PDB ID: 4E9O]. The structure of D8L is shown in gray, while mutations and their labels are colored according to the scheme in the legend.

Further examination of the specific mutations between the immunogenic proteins of VACV and both the MPXV-2022 consensus sequence and the MPXV-CB reference sequence revealed exactly the same sets of mutations for half (4/8) of the proteins. For the remaining proteins, most mutations were still had in common. In these cases, the MPXV-2022 consensus or the MPXV-CB reference sequence carried one or two additional mutations (Table 2). The highest number of common mutations relative to VACV were observed for the H3L and D8L proteins (19 and 17 mutations, respectively). Mapping these mutations onto the reported crystal structures (H3L: [PDB ID 5EJ0] and D8L: [PDB ID 4E9O]) revealed that, for H3L, 7/19 common mutations appear to be exposed (Figure 1A). The unique mutations in MPXV-2022 (A4V) and MPXV-CB (T111I) are also seemingly exposed (Figure 1A); hence, these may be accessible to targeting antibodies and have the potential to affect binding or neutralization. For D8L, 7/17 mutations common in MPXV-2022 and MPXV-CB, as well as the unique mutation in MPXV-CB (A19T), are all exposed (Figure 1B). A subset (4/7) of the common mutations and the unique mutation in MPXV-CB overlaps with the known binding footprints [37] of three D8L-specific VACV NAbs (Figure 2). The potential impact of these mutations on antibody binding and neutralizing capacity remains to be determined. Importantly, for all three antibodies, MPXV-2022 contained no additional mutations in the binding footprints relative to MPXV-CB.

Overall, while some genetic differences were observed between the immunogenic proteins of VACV targeted by NAbs and their orthologs in MPXV-2022, most of these differences were common to MPXV-2022 and MPXV-CB. With >96% genetic similarity within these immunogenic proteins between the reference sequence of VACV and the VACV-based vaccine sequences (Table 3), the humoral immunity induced against MPXV-2002 by these vaccines is anticipated to be similar to that reported against MPXV-CB for the first-generation vaccines.

### 3.2. VACV Proteins and Epitopes Targeted by T Cells Are Largely Conserved in MPXV-2022

Experimental studies have investigated T-cell responses induced by VACV-based vaccines [29,31]. We collated a set of all VACV proteins associated with at least one reported T cell epitope (Methods), revealing a total of 121 proteins. A high degree of genetic similarity was observed between these VACV proteins and their orthologs in MPXV-2022 and MPXV-CB (Figure S3). This would anticipate the significant cross-reactivity of VACV-induced T-cell responses against MPXV-2022 (and MPXV-CB).

**Figure 3:**
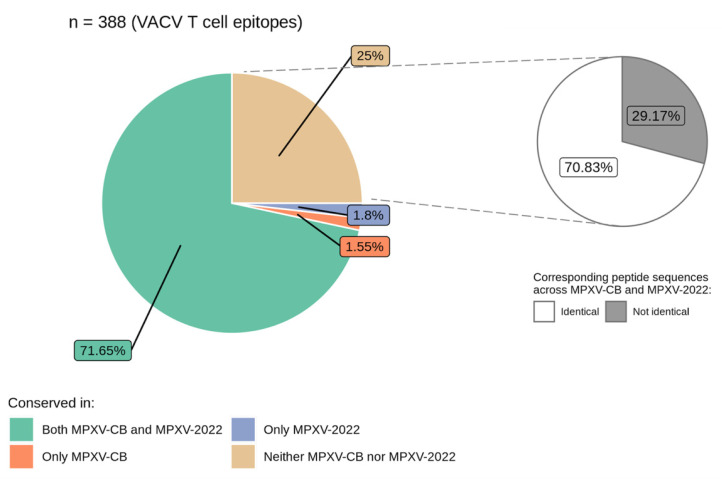
Distribution of the VACV T cell epitopes (n = 388) conserved among MPXV-2022 and MPXV-CB.

Across these 121 proteins, 388 VACV-derived T cell epitopes (197 CD8^+^ and 191 CD4^+^) were identified. Comparing the epitope sequences with the MPXV-2022 consensus and the MPXV-CB reference sequence revealed that 71.6% (278/388) of the epitopes had an exact match in both MPXV-2022 and MPXV-CB. Of the remaining sequences, 1.55% (6/388) differed only in MPXV-2022, 1.8% (7/388) differed only in MPXV-CB, while 25% (97/388) differed in both MPXV-2022 and MPXV-CB (Figure 3 and Table S2). That is, despite high genetic similarity between the VACV proteins and the MPXV-2022 and MPXV-CB orthologs (Figure S3), genetic variation was observed in over one-quarter of the T cell epitopes. The potential impact of this variation on T cell recognition remains to be determined. Importantly, further examination of the 25% of VACV T cell epitopes that differed in both MPXV-2022 and MPXV-CB showed that a large fraction (70%) associated with epitope mutations were identical in both MPXV-2022 and MPXV-CB (Figure 3). Taken together, sequences of 89.2% of the T cell epitopes are identical in both MPXV-2022 and MPXV-CB. Interestingly, for the eight immunogenic proteins targeted by NAbs, a high percentage (93%) of the known VACV T cell epitopes had an exact match in MPXV-2022.

Our analysis, overall, shows that most known VACV T cell epitopes are fully conserved in MPXV-2022, and that while notable variation within VACV T cell epitopes still exists in MPXV-2022, most of this variation is identical to that observed for MPXV-CB. Moreover, >85% of these 388 VACV-derived known T cell epitopes are fully conserved in the Dryvax, ACAM2000, and MVA-BN vaccine sequences (Table S3). Thus, the cellular immunity induced by these VACV-based vaccines against MPXV-2022 is anticipated to be similar to that reported for first-generation vaccines against MPXV-CB.

## 4. Limitations of the Study

There are a number of limitations of our study. Our analysis of anticipated immune responses elicited by VACV-based vaccines against MPXV-2022 is based on the high genetic conservation of the known targets of VACV-elicited antibodies and T cells with MPXV-2022. While the genetic conservation of immune targets among related viruses (e.g., SARS-CoV-1 and SARS-CoV-2 [47], and the dengue and zika viruses [48]) has been shown to be a good predictor of cross-reactive immune responses; experimental studies are required to confirm our findings. Moreover, while we have analyzed known highly immunogenic proteins when assessing neutralizing antibody responses, our analysis of expected T-cell responses does not take into consideration the immunodominance hierarchy of proteins [39], which has been observed in other viruses [49,50]. This may be a confounding factor for the latter analysis, as mutations occurring in a highly immunodominant protein may potentially have a larger impact on T cell immune escape, as compared to mutations occurring in other proteins.

## 5. Discussion

The MPXV-2022 outbreak is the first multi-country spread of monkeypox outside Africa [2,3]. The rapid speed at which the outbreak has spread is concerning. While the MPXV-2022 isolates have been associated with the MPXV-WA clade, these differ on average by 50 SNPs from the closest MPXV-WA sequences, which were collected in 2018–2019 [11]. This number of SNPs is surprising when one compares it to the estimated substitution rate (~1–2 SNPs per genome per year [51,52]) for orthopoxviruses. These genetic differences raise questions about the evolutionary origin of the additional mutations associated with the 2022 outbreak, as well as their potential effects on viral transmission, infectivity, and immune recognition. Research is being pursued to address these questions.

The current study aimed to investigate to what extent the genetic differences observed in the MPXV-2022 outbreak viruses may be expected to impact immune responses induced by VACV-based vaccines. This question is further confounded by the recognition that while the effectiveness of first-generation VACV-based vaccines in humans has been reported for MPXV-CB [4,26], to our knowledge, similar data is not available for MPXV-WA upon immunization with any VACV-based vaccine. Comparing the genetic composition of known targets of VACV-elicited neutralizing antibodies or T cells, either at the epitope level (where available) or the protein level, our analysis demonstrated limited genetic variability among the MPXV-2022 sequences. Moreover, the large majority of corresponding genetic variation in MPXV-2022 was commonly observed in MPXV-CB. Based on this and the knowledge that VACV-based vaccines elicit comparable immune responses [18,20,21,22,23,24,25], it may be anticipated that the currently available VACV-based vaccines (MVA-BN and ACAM2000) will elicit similar humoral and cellular immunity against MPXV-2022, as the first-generation VACV-based vaccines did for MPXV-CB. If this is the case, the efficacy of the current VACV-based vaccines may also be high against MPXV-2022. However, clinical data is required to determine the exact efficacy of these vaccines against MPXV-2022.

## Figures and Tables

**Table 1:** Summary of the VACV proteins known to be targets of NAbs.

No.	VACV Protein	Protein Length (VACV)	PDB ID of 3D Structures	Protein Function [46]	MPXV Protein	Similarity (%)	
						MPXV-CB (Reference Sequence)	MPXV-2022 (Consensus Sequence)
1	A17L	203	-	IMV surface membrane protein, early function in virion morphogenesis	A18	97.54%	98.03%
2	A27L	110	3VOP	IMV surface membrane 14-kDa fusion protein, binds cell surface heparan	A29	94.55%	94.55%
3	A28L	146	-	-	A30	96.58%	96.57%
4	A33R	185	3K7B	EEV envelope glycoprotein, needed for formation of actin-containing microvilli and cell-to-cell spread	A35	96.22%	95.48%
5	B5R	317	-	Palmitylated 42-kDa EEV glycoprotein required for efficient cell spread, complement control	B6	96.53%	96.53%
6	D8L	304	4E9O	IMV surface membrane 32 kDa protein, binds cell surface chondroitin sulfate, IMV adsorption to cell surface	E8	94.08%	94.41%
7	L1R	250	1YPY	Myristylated IMV surface membrane protein	M1	98.40%	98.40%
8	H3L	324	5EJ0	IMV heparan-binding surface membrane protein	H3	93.83%	93.83%

**Table 2:** Genetic comparison of known VACV NAb target proteins with corresponding proteins in MPXV-CB and MPXV-2022. The substitutions are computed with reference to the VACV proteins and using the consensus sequence of the ortholog protein for MPXV-2022.

No.	VACV Protein	Length	Substitutions		
			Only in MPXV-CB	Only in MPXV-2022	Common among MPXV-CB and MPXV-2022
1	A17L	203	T183I	-	Y155F, R165K, T171P, V188I
2	A27L	110	-	-	K27N, A30T, D39Y, E40G, V61I, R74H
3	A28L	146	-	-	G23S, Q110R, V130I, V131A, A137T
4	A33R	177	L59Q	E67K, A88V	A73S, Q117K, L118S, S120E, T127A, I141T
5	B5R	317	-	-	Q50S, S55L, I82V, N87D, A166V, M188I, V233I, I236T, T240S, V283M, V296I
6	D8L	304	A19T	-	N18D, P23T, S65T, L66I, L114I, S118A, L124S, T146M, K163T, V181A, D209E, A246V, R253K, T261A, E272G, F293L, R296Q
7	L1R	250	-	-	L51I, K177R, V242I, M248I
8	H3L	324	T111I	A4V	L17P, P44Q, N48D, V51I, K53N, A63V, N74D, N108K, K117N, V124I, M143I, M168I, N171D, L230M, A233S, N251T, A263V, T265A, A274T

**Table 3:** Similarity of the VACV proteins known to be targets of NAbs with reference sequences for the MVA-BN, ACAM2000, and Dryvax vaccines.

VACV Protein	Similarity (%)		
	MVA-BN	ACAM2000	Dryvax
A17L	99.0	99.5	99.5
A27L	99.1	100.0	100.0
A28L	99.3	100.0	100.0
A33R	98.4	100.0	100.0
B5R	97.2	96.5	97.2
D8L	98.4	98.7	98.7
H3L	98.8	99.1	99.1
LIR	99.6	99.6	99.6

## Data Availability

GISAID acquisition IDs of all Monkeypox sequences, IEDB IDs of all epitope data, and all coding scripts (written in the R language) for reproducing the results are available online as a GitHub repository https://github.com/faraz107/monkeypox-immunogenic-proteins-analysis, accessed on 25 June 2022. The developed in-house pipeline for aligning genome sequences of MPXV-2022 with the VACV reference sequence and for translating to corresponding ortholog proteins is also available at the GitHub repository.

## Supplementary Materials

The following supporting information can be downloaded at: https://www.mdpi.com/article/10.3390/v14091960/s1, Figure S1: Full-genome comparison of the reference sequences for VACV and the three vaccines: MVA-BN, ACAM2000, and Dryvax (consensus sequence); Figure S2: Genetic similarity between VACV proteins targeted by NAbs and their orthologs in 531 MPXV-2022 sequences; Figure S3: Genetic similarity of 121 VACV-specific proteins with known T cell epitopes among MPXV-2022 (red) and MPXV-CB (blue) orthologs; Table S1: List of GISAID accession IDs of the MPXV-2022 sequences analyzed; Table S2: Complete list of experimentally-determined VACV T cell epitopes (n = 388) and their conservation in MPXV-2022 and MPXV-CB; Table S3: Complete list of experimentally-determined VACV T cell epitopes (n = 388) and their conservation in the reference sequences of MVA-BN, ACAM2000, and Dryvax vaccines; Table S4: Acknowledgment table (downloaded from GISAID).
