# Supplementary material for: Vaccinia-Virus-Based Vaccines Are Expected to Elicit Highly Cross-Reactive Immunity to the 2022 Monkeypox Virus
Source: Viruses. 2022 Sep 3;14(9):1960. doi: 10.3390/v14091960 (PMC9506226; doi:10.3390/v14091960)
Supplement: Supplementary file 1 [file viruses-14-01960-s001.zip › Supplementary Table S4.pdf]

We gratefully acknowledge the following Authors from the Originating laboratories responsible for obtaining the specimens, as well as the Submitting laboratories where the genome data were generated and shared via GISAID, on which this research is based.

All Submitters of data may be contacted directly via www.gisaid.org

Authors are sorted alphabetically.

Accession ID	Originating Laboratory	Submitting Laboratory	Authors
EPI_ISL_13052263	Microbiol Genomics and Bioinformatics, Bundeswehr Institute of Microbiology	Microbiol Genomics and Bioinformatics, Bundeswehr Institute of Microbiology	Antwerpen,M.H., Lang,D., Zange,S., Walter,M.C. and Woelfel,R.
EPI_ISL_13052266, EPI_ISL_13052267, EPI_ISL_13052268, EPI_ISL_13052269, EPI_ISL_13052270, EPI_ISL_13052272, EPI_ISL_13052273	Instituto Nacional de Saude Doutor Ricardo Jorge (INSA)	Instituto Nacional de Saude Doutor Ricardo Jorge (INSA)	Joana Isidro, Vítor Borges, Miguel Pinto, Daniel Sobral, João Dourado Santos, Alexandra Nunes, Verónica Mixão, Rita Ferreira, Daniela Santos, Silvia Duarte, Luís Vieira, Maria José Borrego, Sofia Núncio, Isabel Lopes de Carvalho, Ana Pelerito, Rita Cordeiro, João Paulo Gomes
EPI_ISL_13052274	Laboratory of Virology, University Hospitals of Geneva	Laboratory of Virology, University Hospitals of Geneva	Laubscher,F., Chudzinski,V., Schibler,M., Kaiser,L. and Renzoni,A.
EPI_ISL_13052275	IHAP, VIRAL, Universite de Toulouse, INRAE, ENVT	IHAP, VIRAL, Universite de Toulouse, INRAE, ENVT	Croville,G., Walch,M., Guerin,J.-L., Mansuy,J.-M., Pasquier,C. and Izopet,J.
EPI_ISL_13052277	Public Health Virology, Erasmus Medical Centre	Public Health Virology, Erasmus Medical Centre	Oude Munnink,B.B., Boter,M., Wellers,B., Molenkamp,R., Sikkema,R.S. and Koopmans,M.
EPI_ISL_13052278	Research and Evaluation, UKHSA	Research and Evaluation, UKHSA	Osman,K.L., Lewandowski,K.S., Pullan,S.T., Carter,D.P., Crook,J.M., Vipond,R. and Chand,M.
EPI_ISL_13052279, EPI_ISL_13052280, EPI_ISL_13052281	Research and Evaluation, UKHSA	Research and Evaluation, UKHSA	Osman,K.L., Lewandowski,K.S., Carter,D.P., Crook,J.M., Pullan,S.T., Vipond,R. and Chand,M.
EPI_ISL_13052282	Microbiology, Immunology and Transplantation, KU Leuven, Rega Institute	Microbiology, Immunology and Transplantation, KU Leuven, Rega Institute	Vanmechelen,B., Wawina-Bokalanga,T., Logist,A.-S., Sinnesael,R., Ysebaert,L., Verlinden,J., Bloemen,M. and Maes,P.
EPI_ISL_13052283	Microbiology, Immunology and Transplantation, KU Leuven, Rega Institute	Microbiology, Immunology and Transplantation, KU Leuven, Rega Institute	Wawina-Bokalanga,T., Vanmechelen,B., Logist,A.-S., Sinnesael,R., Ysebaert,L., Verlinden,J., Bloemen,M. and Maes,P.
EPI_ISL_13052284	Microbiology, Hospital Universitari Germans Trias i Pujol	Microbiology, Hospital Universitari Germans Trias i Pujol	Martinez-Puchol,S., Coello,A., Bordoy,A.E., Soler,L., Panisello,D., Gonzalez-Gomez,S., Clara,G., Paris de Leon,A., Not,A., Hernandez,A., Bofill-Mas,S., Saludes,V., Blanco,I., Martro,E. and Cardona,P.-J.
EPI_ISL_13052287	Virology, GENomique EPIdemiologique des maladies Infectieuses	Virology, GENomique EPIdemiologique des maladies Infectieuses	unknown
EPI_ISL_13052288	Department of Health, Utah Public Health Laboratory	Department of Health, Utah Public Health Laboratory	Young,E.L., Hergert,J. and Oakeson,K.F.
EPI_ISL_13052289	Division of High-Consequence Pathogens and Pathology, Centers for Disease Control and Prevention	Division of High-Consequence Pathogens and Pathology, Centers for Disease Control and Prevention	Gigante,C.M., Smole,S., Seabolt,M.H., Wilkins,K., McCollum,A., Hutson,C., Davidson,W., Rao,A., Brown,C. and Li,Y.
EPI_ISL_13052290	Laboratory for Diagnostics of Zoonoses and WHO Centre, Institute of Microbiology and Immunology, Faculty of Medicine, University of Ljubljana	Laboratory for Diagnostics of Zoonoses and WHO Centre, Institute of Microbiology and Immunology, Faculty of Medicine, University of Ljubljana	Zakotnik,S., Vlaj,D., Suljic,A., Zorec,T.M., Korva,M., Poljak,M. and Avsic Zupanc,T.
EPI_ISL_13052291	Laboratory for Diagnostics of Zoonoses and WHO Centre, Institute of Microbiology and Immunology, Faculty of Medicine, University of Ljubljana	Laboratory for Diagnostics of Zoonoses and WHO Centre, Institute of Microbiology and Immunology, Faculty of Medicine, University of Ljubljana	Zakotnik,S., Vlaj,D., Suljic,A., Zorec,T.M., Korva,M. and Avsic Zupanc,T.
EPI_ISL_13052292	Victorian Infectious Diseases Reference Laboratory, Doherty Institute	Victorian Infectious Diseases Reference Laboratory, Doherty Institute	Hammerschlag,Y., MacLeod,G., Papadakis,G., Adan-Sanchez,A., Druce,J.D., Williamson,D.A., Cheng,A.C. and McMahon,J.H.
EPI_ISL_13052293, EPI_ISL_13052294	Centre for Biological Threats, Highly Pathogenic Viruses, Robert Koch Institute	Centre for Biological Threats, Highly Pathogenic Viruses, Robert Koch Institute	Brinkmann,A., Kohl,C., Uddin,S., Pape,K., Schrick,L., Michel,J., Schaade,L. and Nitsche,A.
EPI_ISL_13052295	SC (UCO) Igiene e Sanità Pubblica, ASUGI, Trieste	Genomics and Epigenomics, AREA Science Park	Licastro,D., DeGasperi,M., Negri,C., Piscianz,E., Koncan,R., Dal Monego,S., Segat,L. and D'Agaro,P.
EPI_ISL_13056892, EPI_ISL_13056893, EPI_ISL_13056894, EPI_ISL_13056895, EPI_ISL_13056896, EPI_ISL_13056897, EPI_ISL_13056899, EPI_ISL_13056900, EPI_ISL_13056901, EPI_ISL_13056902, EPI_ISL_13056903, EPI_ISL_13056904, EPI_ISL_13056905, EPI_ISL_13056906, EPI_ISL_13056907, EPI_ISL_13056908, EPI_ISL_13056909			
see above	Instituto Nacional de Saude Doutor Ricardo Jorge (INSA)	Instituto Nacional de Saude Doutor Ricardo Jorge (INSA)	Joana Isidro, Vítor Borges, Miguel Pinto, Daniel Sobral, João Dourado Santos, Alexandra Nunes, Verónica Mixão, Rita Ferreira, Daniela Santos, Silvia Duarte, Luís Vieira, Maria José Borrego, Sofia Núncio, Isabel Lopes de Carvalho, Ana Pelerito, Rita Cordeiro, João Paulo Gomes
EPI_ISL_13056910	Biochemistry and Molecular Genetics, Israel Institute for Biological Research	Biochemistry and Molecular Genetics, Israel Institute for Biological Research	Israeli,O., Guedj-Dana,Y., Lazar,S., Shifman,O., Erez,N., Weiss,S., Paran,N., Israely,T., Schuster,O., Zvi,A., Beth-Din,A. and Cohen Gihon,I.
EPI_ISL_13089461	Hospital General Universitario Gregorio Marañón	Hospital General Universitario Gregorio Marañón	Sergio Buenestado Serrano, Rosalía Palomino Cabrera, Daniel Peñas Utrilla, Jorge Rodríguez-Grande, Laura Pérez-Lago, Cristina Rodriguez-Grande, Marta Herranz Martin, Julia Suárez, Pilar Catalán, Patricia Muñoz, Darío García de Viedma
EPI_ISL_13094227	Division of High-Consequence Pathogens and Pathology, Centers for Disease Control and Prevention	Division of High-Consequence Pathogens and Pathology, Centers for Disease Control and Prevention	Gigante,C.M., Lee,P., Seabolt,M.H., Wilkins,K., McCollum,A., Hutson,C., Davidson,W., Rao,A., Mendoza,R. and Li,Y.
EPI_ISL_13096615	Division of High-Consequence Pathogens and Pathology, Centers for Disease Control and Prevention	Division of High-Consequence Pathogens and Pathology, Centers for Disease Control and Prevention	Gigante,C.M., Griffin-Thomas,L.A., Seabolt,M.H., Wilkins,K., McCollum,A., Hutson,C., Davidson,W., Rao,A., Crain,J. and Li,Y.
EPI_ISL_13100618	Division of High-Consequence Pathogens and Pathology, Centers for Disease Control and Prevention	Division of High-Consequence Pathogens and Pathology, Centers for Disease Control and Prevention	Gigante,C.M., Ventura,J., Seabolt,M.H., Wilkins,K., McCollum,A., Hutson,C., Davidson,W., Rao,A., Nash,J. and Li,Y.
EPI_ISL_13100619	Division of High-Consequence Pathogens and Pathology, Centers for Disease Control and Prevention	Division of High-Consequence Pathogens and Pathology, Centers for Disease Control and Prevention	Gigante,C.M., Lee,P., Seabolt,M.H., Wilkins,K., McCollum,A., Hutson,C., Davidson,W., Rao,A., Mendoza,R. and Li,Y.
EPI_ISL_13100620	Division of High-Consequence Pathogens and Pathology, Centers for Disease Control and Prevention	Division of High-Consequence Pathogens and Pathology, Centers for Disease Control and Prevention	Gigante,C.M., Atkinson,A., Seabolt,M.H., Wilkins,K., McCollum,A., Hutson,C., Davidson,W., Rao,A., Murray,J. and Li,Y.
EPI_ISL_13100719	Division of High-Consequence Pathogens and Pathology, Centers for Disease Control and Prevention	Division of High-Consequence Pathogens and Pathology, Centers for Disease Control and Prevention	Gigante,C.M., Ventura,J., Seabolt,M.H., Wilkins,K., McCollum,A., Hutson,C., Davidson,W., Rao,A., Nash,J. and Li,Y.
EPI_ISL_13106454	Hospital General Universitario Gregorio Marañón	Hospital General Universitario Gregorio Marañón	Sergio Buenestado Serrano, Rosalía Palomino Cabrera, Daniel Peñas Utrilla, Jorge Rodríguez-Grande, Pedro Sola Campoy, Laura Pérez-Lago, Cristina Rodriguez-Grande, Marta Herranz Martin, Julia Suárez, Pilar Catalán, Patricia Muñoz, Darío García de Viedma
EPI_ISL_13117291	Centre for Biological Threats, Highly Pathogenic Viruses, Robert Koch Institute	Centre for Biological Threats, Highly Pathogenic Viruses, Robert Koch Institute	Brinkmann,A., Kohl,C., Uddin,S., Pape,K., Schrick,L., Michel,J., Jessen,H., Schaade,L. and Michel,A.
EPI_ISL_13117292, EPI_ISL_13117293	Centre for Biological Threats, Highly Pathogenic Viruses, Robert Koch Institute	Centre for Biological Threats, Highly Pathogenic Viruses, Robert Koch Institute	Brinkmann,A., Kohl,C., Uddin,S., Pape,K., Schrick,L., Michel,J., Stocker,H., Schaade,L. and Nitsche,A.

EPI_ISL_13117294, EPI_ISL_13117295, EPI_ISL_13117296, EPI_ISL_13117297, EPI_ISL_13117298	Centre for Biological Threats, Highly Pathogenic Viruses, Robert Koch Institute	Centre for Biological Threats, Highly Pathogenic Viruses, Robert Koch Institute	Brinkmann,A., Kohl,C., Uddin,S., Pape,K., Schrick,L., Michel,J., Schaade,L. and Nitsche,A.
EPI_ISL_13148263, EPI_ISL_13148264, EPI_ISL_13148265	Centre for Biological Threats, Highly Pathogenic Viruses, Robert Koch Institute	Centre for Biological Threats, Highly Pathogenic Viruses, Robert Koch Institute	Brinkmann,A., Kohl,C., Uddin,S., Pape,K., Schrick,L., Michel,J., Jessen,H., Schaade,L. and Nitsche,A.
EPI_ISL_13148266, EPI_ISL_13148267, EPI_ISL_13148268, EPI_ISL_13148269	Centre for Biological Threats, Highly Pathogenic Viruses, Robert Koch Institute	Centre for Biological Threats, Highly Pathogenic Viruses, Robert Koch Institute	Brinkmann,A., Kohl,C., Uddin,S., Pape,K., Schrick,L., Michel,J., Stocker,H., Schaade,L. and Nitsche,A.
EPI_ISL_13148270, EPI_ISL_13148271, EPI_ISL_13148272, EPI_ISL_13148273, EPI_ISL_13148274, EPI_ISL_13148275, EPI_ISL_13148276	Centre for Biological Threats, Highly Pathogenic Viruses, Robert Koch Institute	Centre for Biological Threats, Highly Pathogenic Viruses, Robert Koch Institute	Brinkmann,A., Kohl,C., Uddin,S., Pape,K., Schrick,L., Michel,J., Schaade,L. and Nitsche,A.
EPI_ISL_13191438	Instituto de Infectologia Emilio Ribas	Instituto Adolfo Lutz Strategic Laboratory	Claudio Tavares Sacchi, Karoline Rodrigues Campos, Marlon Benedito Nascimento Santos, Alex Domingos Reis, Ariadne Ferreira Amarante, Adriano Abbud, Adriana Bugno, Walkiria Delnoro Almeida Prado, Regiane Cardoso de Paula
EPI_ISL_13194516	Alberta Precision Laboratories	Alberta Precision Laboratories	Matthew Croxen, Ashwin Deo, Paul Dieu, Xiaoli Dong, Kara Gill, David Granger, Christina Ferrato, Vanipriyadarsini Ikkurti, Jamil Kanji, Petya Koleva, Vincent Li, Colin Lloyd, Tarah Lynch, Raymond Ma, Kanti Pabbaraju, Silas Rotich, Hilary Sergeant, Steven Shideler, Todd Skitsko, Sandy Shokopoles, Graham Tipples, Johanna Thayer, Anita Wong
EPI_ISL_13234112	Laboratório Central de Saúde Pública do Estado do Rio Grande do Sul	Instituto Adolfo Lutz Strategic Laboratory	Claudio Tavares Sacchi, Karoline Rodrigues Campos, Adriano Abbud, Adriana Bugno
EPI_ISL_13242738	Hospital General Universitario Gregorio Marañón	Hospital General Universitario Gregorio Marañón	Sergio Buenestado Serrano, Rosalía Palomino Cabrera, Daniel Peñas Utrilla, Jorge Rodríguez-Grande, Pedro Sola Campoy, Laura Pérez-Lago, Cristina Rodriguez-Grande, Marta Herranz Martin, Julia Suárez, Pilar Catalán, Patricia Muñoz, Darío García de Viedma
EPI_ISL_13244349	Erasmus Medical Center Department of Virology	Erasmus Medical Center Department of Virology	Bas Oude Munnink, Marjan Boter, Babette Weller, Richard Molenkamp, Janette Rahamat-Langendoen, Reina Sikkema, Marion Koopmans
EPI_ISL_13251120	Laboratory of Virology, INMI Lazzaro Spallanzani IRCCS	Laboratory of Virology, INMI Lazzaro Spallanzani IRCCS	Giombini,E., Gruber,C.E.M., Rueca,M., Gramigna,G., VIta,S., Carletti,F., D'Abramo,A., Lapa,D., Puro,V., Fabeni,L., Butera,O., Colavita,F., Meschi,S., Matusali,G., Specchiarello,E., Vairo,F., Vaia,F., Garbuglia,A.R., Nicastri,E., Antinori,A., GIrardi,E. and Maggi,F.
EPI_ISL_13251157	checkin Zollhaus	Institute of Medical Virology, University of Zurich	Verena Kufner, Gabriela Ziltener, Maryam Zaheri, Stefan Schmutz, Annette Audigé, Odette Bernasconi, Kevin Steiner, Jon Huder, Cyril Shah, Riccarda Capaul, Guido Bloemberg, Jürg Böni, Michael Huber, Alexandra Trkola
EPI_ISL_13251584	Division of Infectious Diseases, University Hospital Zürich	Institute of Medical Virology, University of Zurich	Verena Kufner, Gabriela Ziltener, Maryam Zaheri, Stefan Schmutz, Annette Audigé, Odette Bernasconi, Kevin Steiner, Jon Huder, Cyril Shah, Riccarda Capaul, Guido Bloemberg, Jürg Böni, Michael Huber, Alexandra Trkola
EPI_ISL_13251723	checkin Zollhaus	Institute of Medical Virology, University of Zurich	Verena Kufner, Gabriela Ziltener, Maryam Zaheri, Stefan Schmutz, Annette Audigé, Odette Bernasconi, Kevin Steiner, Jon Huder, Cyril Shah, Riccarda Capaul, Guido Bloemberg, Jürg Böni, Michael Huber, Alexandra Trkola
EPI_ISL_13269478	Alberta Precision Laboratories	Alberta Precision Laboratories	Matthew Croxen, Ashwin Deo, Paul Dieu, Xiaoli Dong, Kara Gill, David Granger, Christina Ferrato, Vanipriyadarsini Ikkurti, Jamil Kanji, Petya Koleva, Vincent Li, Colin Lloyd, Tarah Lynch, Raymond Ma, Kanti Pabbaraju, Silas Rotich, Hilary Sergeant, Steven Shideler, Todd Skitsko, Sandy Shokopoles, Graham Tipples, Johanna Thayer, Anita Wong
EPI_ISL_13270980	Instituto de Infectologia Emilio Ribas	Imperial College London, School of Public Health	Claro,I.M., de Lima,E.L., Romano,C.M., Candido,D.S., Lindoso,J.A.L., Barra,L.A.C., Borges,L.M.S., Medeiros,L.A., Tomishige,M.Y.S., Ramundo,M.S., Moutinho,T., da Silva,A.J.D., Rodrigues,C.C.M., de Azevedo,L.C.F., Villas-Boas,L.S., da Silva,C.A.M., Coletti,T.M., O'Toole,A., Quick,J., Loman,N., Rambaut,A., Faria,N.R., Figueiredo-Mello,C. and Sabino,E.C.
EPI_ISL_13302316	Laboratory of Clinical Microbiology, Virology and Bioemergencies. ASST-Fatebenefratelli-Sacco, L.Sacco University Hospital	Army Medical and Veterinary Research Center	Silvia Fillo, Riccardo De Sanctis, Giovanni Faggioni, Andrea Ciammaruconi, Anna Anselmo, Vanessa Vera Fain, Simone Di Sabatino, Francesco Giordani, Antonella Fortunato, Rossella Brandi, Giulia Campoli, Marzia Cavalli, Anella Monte, Martina Lipari, Maria Di Spirito, Giorgia Grilli, Silvia Chimienti, Giandomenico Cerreto, Filippo Molinari, Giancarlo Petralito, Davide Mileto, Valeria Micheli, Maria Rita Gismondo, Florigio Lista
EPI_ISL_13304977	National Public Health Center, National Biosafety Laboratory	National Public Health Center, National Biosafety Laboratory	Judit Henczkó, Dániel Déri, Lili Jármi, Bernadett Pályi, Zoltán Kis,
EPI_ISL_13308117, EPI_ISL_13308118, EPI_ISL_13308119, EPI_ISL_13308121, EPI_ISL_13308122, EPI_ISL_13308124, EPI_ISL_13308125, EPI_ISL_13308127, EPI_ISL_13308129, EPI_ISL_13308131, EPI_ISL_13308133, EPI_ISL_13308135, EPI_ISL_13308137, EPI_ISL_13308139, EPI_ISL_13308140, EPI_ISL_13308142, EPI_ISL_13308144, EPI_ISL_13308145, EPI_ISL_13308146, EPI_ISL_13308147, EPI_ISL_13308148, EPI_ISL_13308150, EPI_ISL_13308151, EPI_ISL_13308153, EPI_ISL_13308155, EPI_ISL_13308157			
see above	Centre for Biological Threats, Highly Pathogenic Viruses, Robert Koch Institute	Centre for Biological Threats, Highly Pathogenic Viruses, Robert Koch Institute	Brinkmann,A., Kohl,C., Uddin,S., Pape,K., Schrick,L., Michel,J., Schaade,L. and Nitsche,A.
EPI_ISL_13308158, EPI_ISL_13308160	IRBA Research Institute Biomédicale Des Armées	IRBA Research Institute Biomédicale Des Armées	Jarjaval,F., Nolent,F., Criqui,A., Chapus,C., Lamer,O., Ferraris,O. and Gorge,O.
EPI_ISL_13308162, EPI_ISL_13308163, EPI_ISL_13308165, EPI_ISL_13308167	Laboratory for Diagnostics of Zoonoses and WHO Centre, Institute of Microbiology and Immunology, Faculty of Medicine, University of Ljubljana	Laboratory for Diagnostics of Zoonoses and WHO Centre, Institute of Microbiology and Immunology, Faculty of Medicine, University of Ljubljana	Zakotnik,S., Vlaj,D., Suljic,A., Zorec,T.M., Korva,M., Poljak,M. and Avsic Zupanc,T.
EPI_ISL_13314740	Laboratorio de Vigilancia em Saude de Vinhedo	Instituto Adolfo Lutz Strategic Laboratory	Claudio Tavares Sacchi, Karoline Rodrigues Campos, Adriano Abbud, Adriana Bugno
EPI_ISL_13331598	Department for Virology, Molecular Biology and Genome Research, R. G. Lugar Center for Public Health Research, National Center for Disease Control and Public Health (NCDC) of Georgia	Department for Virology, Molecular Biology and Genome Research, R. G. Lugar Center for Public Health Research, National Center for Disease Control and Public Health (NCDC) of Georgia	Giorgi Tomashvili, Salome Javashvili, Meri Pantsulaia, Gvantsa Brachveli, Ana Papkiauri, Gvantsa Chanturia, Adam Kotorashvili, Maia Alkhazashvili, Khatuna Zakhashvili, Paata Imnadze, Amiran Gamkrelidze.
EPI_ISL_13331712	Laboratory of Virology, INMI Lazzaro Spallanzani IRCCS	Laboratory of Virology, INMI Lazzaro Spallanzani IRCCS	Rueca,M., Giombini,E., Gruber,C.E.M., Gramigna,G., Mazzotta,V., Carletti,F., Lapa,D., Pittalis,S., Puro,V., Fabeni,L., Butera,O., Colavita,F., Meschi,S., Matusali,G., Specchiarello,E., Vairo,F., Vaia,F., Nicastri,E., Antinori,A., Girardi,E. and Maggi,F.
EPI_ISL_13331713	Laboratory of Virology, INMI Lazzaro Spallanzani IRCCS	Laboratory of Virology, INMI Lazzaro Spallanzani IRCCS	Gramigna,G., Giombini,E., Gruber,C.E.M., Rueca,M., Carletti,F., Cicalini,S., Lapa,D., Puro,V., Marani,A., Fabeni,L., Butera,O., Colavita,F., Meschi,S., Matusali,G., Rivano Capparuccia,M., Specchiarello,E., Vairo,F., Vaia,F., Nicastri,E., Antinori,A., Girardi,E. and Maggi,F.
EPI_ISL_13331714	Department of Virology, Faculty of Medicine, University of Helsinki, Hartmaninkatu 3	Department of Virology, Faculty of Medicine, University of Helsinki, Hartmaninkatu 3	Kant,R., Smura,T., Vauhkonen,H. and Vapalahti,O.
EPI_ISL_13331715	Department of Virology, Faculty of Medicine, University of Helsinki, Hartmaninkatu 3	Department of Virology, Faculty of Medicine, University of Helsinki, Hartmaninkatu 3	Kant,R., Smura,T., Vauhkonen,H., Vapalahti,O. and Sironen,T.
EPI_ISL_13331717	Genomics Division, Instituto Tecnologico y de Energias Renovables (ITER), Poligono Industrial de Granadilla	Genomics Division, Instituto Tecnologico y de Energias Renovables (ITER), Poligono Industrial de Granadilla,	Alcoba-Florez,J., Munoz-Barrera,A., Ciuffreda,L., Rodriguez-Perez,H., Rubio-Rodriguez,L.A., Gil-Campesino,H., Garcia-Martinez de Artola,D., Inigo-Campos,A., Diez-Gil,O., Gonzalez-Montelongo,R., Valenzuela-Fernandez,A., Lorenzo-Salazar,J.M. and Flores,C.
EPI_ISL_13338028	Clinical Virology Unit, Department of Clinical Sciences, Institute of Tropical Medicine of Antwerp	Clinical Virology Unit, Department of Clinical Sciences, Institute of Tropical Medicine of Antwerp	Antonio Mauro Rezende*, Tessa de Block*, Sandra Coppens, Eric Florence, Maartje van Frankenhuijsen, Stefanie Bracke, Isabel Brosius, Laurens Liesenborghs, Patrick Soentjens, Kevin Ariën, Marjan Van Esbroeck, Philippe Selhorst*, Koen Vercauteren* *equal contribution
EPI_ISL_13339105	Microbiology Service, Hospital Universitario Clinico San Cecilio, Granada	Microbiology Service, Hospital Universitario Clinico San Cecilio, Granada	Chueca N, de Salazar A, Viñuela L, Fuentes A, Casimiro-Soriguer CS, Perez-Florido J, Dopazo J, Garcia F
EPI_ISL_13342823	Clinical Virology Unit, Department of Clinical Sciences, Institute of Tropical Medicine of Antwerp	Clinical Virology Unit, Department of Clinical Sciences, Institute of Tropical Medicine of Antwerp	Philippe Selhorst, Antonio Mauro Rezende, Tessa de Block, Sandra Coppens, Eric Florence, Isabel Brosius, Laurens Liesenborghs, Kevin Ariën, Marjan Van Esbroeck, Chris Kenyon, Koen Vercauteren
EPI_ISL_13343634	Instituto de Infectologia Emilio Ribas	Instituto Adolfo Lutz Strategic Laboratory	Claudio Tavares Sacchi, Karoline Rodrigues Campos, Adriano Abbud, Adriana Bugno
EPI_ISL_13343697	Fleury Medicina Dignóstica	Instituto Adolfo Lutz Strategic Laboratory	Claudio Tavares Sacchi, Karoline Rodrigues Campos, Adriano Abbud, Adriana Bugno
EPI_ISL_13343718	Hospital Santa Ignes	Instituto Adolfo Lutz Strategic Laboratory	Claudio Tavares Sacchi, Karoline Rodrigues Campos, Adriano Abbud, Adriana Bugno

EPI_ISL_13351002	B.C. Centre for Disease Control Public Health Laboratory	B.C. Centre for Disease Control Public Health Laboratory	John Tyson, Tracy Lee, Anthea Lam, Josh Quick, Agatha Jassem, Natalie Prystajecky, Linda Hoang, Inna Sekirov, Catherine Hogn, Frankie Tsang, Mel Krajden
EPI_ISL_13362760, EPI_ISL_13362764	Laboratorio di Epidemiologia Molecolare e Sanità Pubblica-Policlinico Bari	Istituto Zooprofilattico Sperimentale della Puglia e della Basilicata	Parisi A, Simone D, Capozzi L, Del Sambro L, Bianco A, Chironna M, Loconsole D, Sallustio F, Galante D, Pace L, Manzulli V, Fasanella A.
EPI_ISL_13363142	Hospital Universitari Vall d'Hebron	Hospital Universitari Vall d'Hebron	Maria Piñana, Cristina Andrés, Alejandra González-Sánchez, Damir Garcia-Cehic, Ariadna Rando, Juliana Esperalba, Maria Gema Codina, Maria Carmen Martin, Carla Castillo, Karen García, Rodrigo Vásquez, Maria Piquer, Tomàs Pumarola, Josep Quer, Andrés Antón
EPI_ISL_13374487	National Public Health Center, National Biosafety Laboratory	National Public Health Center, National Biosafety Laboratory	Judit Henczkó, Dániel Déri, Fruzsina Petrovay, Lili Jármi, Bernadett Pályi, Eszter Balla, Zoltán Kis
EPI_ISL_13408799, EPI_ISL_13408801, EPI_ISL_13408803	Public Health Agency of Canada, National Microbiology Laboratory	Public Health Agency of Canada, National Microbiology Laboratory	Knox,N., Hole,D., Duggan,A., Yadav,C., Haidl,E., Chapel,M., Graham,M., Domselaar,G.V., Jolly,G., Audet,J., Fernando,L., Antonation,K., Hagan,M., Griffiths,E., Leung,A., Safronetz,D., Eshaghi,A., Gubbay,J.B., Hasso,M., Marchand-Austin,A., Olsha,R. and Patel,S.N.
EPI_ISL_13408805, EPI_ISL_13408807, EPI_ISL_13408809, EPI_ISL_13408811, EPI_ISL_13408813, EPI_ISL_13408815, EPI_ISL_13408817, EPI_ISL_13408819, EPI_ISL_13408821, EPI_ISL_13408823, EPI_ISL_13408825, EPI_ISL_13408827, EPI_ISL_13408829, EPI_ISL_13408831, EPI_ISL_13408833, EPI_ISL_13408835			
see above	Public Health Agency of Canada, National Microbiology Laboratory	Public Health Agency of Canada, National Microbiology Laboratory	ncknox
EPI_ISL_13408837, EPI_ISL_13408839, EPI_ISL_13408841, EPI_ISL_13408843, EPI_ISL_13408845, EPI_ISL_13408847, EPI_ISL_13408849, EPI_ISL_13408851, EPI_ISL_13408853, EPI_ISL_13408855, EPI_ISL_13408857, EPI_ISL_13408859, EPI_ISL_13408861			
see above	Public Health Agency of Canada, National Microbiology Laboratory	Public Health Agency of Canada, National Microbiology Laboratory	Knox,N., Duggan,A., Yadav,C., Hole,D., Haidl,E., Chapel,M., Jolly,G., Domselaar,G.V., Antonation,K., Leung,A., Fernando,L., Audet,J., Hagan,M., Graham,M., Griffiths,E., Safronetz,D., Charest,H., Levade,I. and Fafard,J.
EPI_ISL_13409177, EPI_ISL_13409178, EPI_ISL_13409179, EPI_ISL_13409180, EPI_ISL_13409181	Viral Genomics and Bioinformatics, MRC University of Glasgow Centre for Virus Research	Viral Genomics and Bioinformatics, MRC University of Glasgow Centre for Virus Research	Filipe,A., Tong,L., Vattipally,S.B., Maclean,A., Gunson,R., Holden,M.T.G., Barr,D., Ho,A., Palmarini,M., Rambaut,A., Robertson,D.L. and Thomson,E.C.
EPI_ISL_13411153, EPI_ISL_13411154, EPI_ISL_13411155, EPI_ISL_13411156, EPI_ISL_13411157, EPI_ISL_13411158	Centre for Biological Threats, Highly Pathogenic Viruses, Robert Koch Institute	Centre for Biological Threats, Highly Pathogenic Viruses, Robert Koch Institute	Brinkmann,A., Kohl,C., Uddin,S., Pape,K., Schrick,L., Michel,J., Schaade,L. and Nitsche,A.
EPI_ISL_13411159, EPI_ISL_13411160, EPI_ISL_13411161, EPI_ISL_13411162	Centre for Biological Threats, Highly Pathogenic Viruses, Robert Koch Institute	Centre for Biological Threats, Highly Pathogenic Viruses, Robert Koch Institute	Brinkmann,A., Kohl,C., Uddin,S., Pape,K., Schrick,L., Michel,J., Stocker,H., Schaade,L. and Nitsche,A.
EPI_ISL_13411163, EPI_ISL_13411164, EPI_ISL_13411165	Centre for Biological Threats, Highly Pathogenic Viruses, Robert Koch Institute	Centre for Biological Threats, Highly Pathogenic Viruses, Robert Koch Institute	Brinkmann,A., Kohl,C., Uddin,S., Pape,K., Schrick,L., Michel,J., Schaade,L. and Nitsche,A.
EPI_ISL_13411166, EPI_ISL_13411167	Centre for Biological Threats, Highly Pathogenic Viruses, Robert Koch Institute	Centre for Biological Threats, Highly Pathogenic Viruses, Robert Koch Institute	Brinkmann,A., Kohl,C., Uddin,S., Pape,K., Schrick,L., Michel,J., Jessen,H., Schaade,L. and Nitsche,A.
EPI_ISL_13411168	Centre for Biological Threats, Highly Pathogenic Viruses, Robert Koch Institute	Centre for Biological Threats, Highly Pathogenic Viruses, Robert Koch Institute	Brinkmann,A., Kohl,C., Uddin,S., Pape,K., Schrick,L., Michel,J., Pfaefflin,F., Schaade,L. and Nitsche,A.
EPI_ISL_13436658	Coordenadoria de Vigilancia em Saude - Sao Paulo	Instituto Adolfo Lutz Strategic Laboratory	Claudio Tavares Sacchi, Karoline Rodrigues Campos, Ariadne Fereira Amarante, Adriano Abbud, Adriana Bugno
EPI_ISL_13436792	Hospital Santa Ignes	Instituto Adolfo Lutz Strategic Laboratory	Claudio Tavares Sacchi, Karoline Rodrigues Campos, Adriano Abbud, Adriana Bugno
EPI_ISL_13437056	Hosp. Alemao Oswaldo Cruz	Instituto Adolfo Lutz Strategic Laboratory	Claudio Tavares Sacchi, Karoline Rodrigues Campos, Ariadne Ferreira Amarante, Adriano Abbud, Adriana Bugno
EPI_ISL_13445553	Laboratory for Diagnostics of Zoonoses and WHO Centre, Institute of Microbiology and Immunology, Faculty of Medicine, University of Ljubljana	Laboratory for Diagnostics of Zoonoses and WHO Centre, Institute of Microbiology and Immunology, Faculty of Medicine, University of Ljubljana	Zakotnik,S., Vlaj,D., Suljic,A., Zorec,T.M., Korva,M., Poljak,M. and Avsic Zupanc,T.
EPI_ISL_13449965, EPI_ISL_13449966	Hospital Universitario La Paz, Microbiology	Hospital Universitario La Paz, Microbiology	de la Hoz-Sanchez,B., Lopez-Ortiz,M., Gutierrez-Arroyo,A., Roces-Alvarez,P., Lazaro-Peona,F., Dahdouh,E., Bloise,I., Garcia-Rodriguez,J. and Mingorance,J.
EPI_ISL_13459346	CRT-DST-AIDS	Instituto Adolfo Lutz Strategic Laboratory	Claudio Tavares Sacchi, Karoline Rodrigues Campos, Ariadne Ferreira Amarante, Adriano Abbud, Adriana Bugno
EPI_ISL_13459347, EPI_ISL_13459482, EPI_ISL_13459483	Instituto de Infectologia Emilio Ribas	Instituto Adolfo Lutz Strategic Laboratory	Claudio Tavares Sacchi, Karoline Rodrigues Campos, Ariadne Ferreira Amarante, Adriano Abbud, Adriana Bugno
EPI_ISL_13466447, EPI_ISL_13466448, EPI_ISL_13466449, EPI_ISL_13466450, EPI_ISL_13466451, EPI_ISL_13466452, EPI_ISL_13466453, EPI_ISL_13466455, EPI_ISL_13466456, EPI_ISL_13466457, EPI_ISL_13466458, EPI_ISL_13466459, EPI_ISL_13466460, EPI_ISL_13466461, EPI_ISL_13466462, EPI_ISL_13466464, EPI_ISL_13466465			
see above	Department of Infectious Diseases, National Institute of Health Doutor Ricardo Jorge, Portugal (INSA)	Department of Infectious Diseases, National Institute of Health Doutor Ricardo Jorge, Portugal (INSA)	Isidro,J., Borges,V., Pinto,M., Sobral,D., Santos,J., Nunes,A., Mixao,V., Ferreira,R., Santos,D., Duarte,S., Vieira,L., Borrego,M.J., Nuncio,S., Lopes de Carvalho,I., Pelerito,A., Cordeiro,R., Gomes,J.P.
EPI_ISL_13472080	National Institute of Public Health NIH - NRI	National Institute of Public Health NIH - NRI	Wokowicz Tomasz, Zacharczuk Katarzyna, Gierczyski Rafa
EPI_ISL_13472250	Medical University of Vienna Center for Virology	Medical University of Vienna Center for Virology	Jeremy V. Camp, Monika Redlberger-Fritz, Stephan W. Aberle
EPI_ISL_13483155, EPI_ISL_13483157, EPI_ISL_13483159, EPI_ISL_13483161, EPI_ISL_13483162, EPI_ISL_13483163, EPI_ISL_13483164	Centre for Biological Threats, Highly Pathogenic Viruses, Robert Koch Institute	Centre for Biological Threats, Highly Pathogenic Viruses, Robert Koch Institute	Brinkmann,A., Kohl,C., Uddin,S., Pape,K., Schrick,L., Michel,J., Jessen,H., Schaade,L. and Nitsche,A.
EPI_ISL_13483165, EPI_ISL_13483167, EPI_ISL_13483168, EPI_ISL_13483170, EPI_ISL_13483171, EPI_ISL_13483173, EPI_ISL_13483175, EPI_ISL_13483177, EPI_ISL_13483178, EPI_ISL_13483180, EPI_ISL_13483182, EPI_ISL_13483183, EPI_ISL_13483185, EPI_ISL_13483187, EPI_ISL_13483188, EPI_ISL_13483190, EPI_ISL_13483191, EPI_ISL_13483193, EPI_ISL_13483195, EPI_ISL_13483196, EPI_ISL_13483198, EPI_ISL_13483200, EPI_ISL_13483201, EPI_ISL_13483203, EPI_ISL_13483205, EPI_ISL_13483206, EPI_ISL_13483208			
see above	Centre for Biological Threats, Highly Pathogenic Viruses, Robert Koch Institute	Centre for Biological Threats, Highly Pathogenic Viruses, Robert Koch Institute	Brinkmann,A., Kohl,C., Uddin,S., Pape,K., Schrick,L., Michel,J., Schaade,L. and Nitsche,A.
EPI_ISL_13484458	Laboratorio de Enterovirus, Instituto Oswaldo Cruz, Fiocruz	Instituto Oswaldo Cruz FIOCRUZ - Laboratory of Respiratory Viruses and Measles (LVRS)	Paola Resende, Elisa Cavalcante Pereira, Bruna Mendonça da Silva, Jéssica Graça Macedo de Carvalho, Larissa Macedo Pinto, Victor Guimaraes, Marilda Siqueira, Renan da Silva Faustino, Marilia Santini, Edson Elias da Silva on behalf of the Fiocruz Genomic Surveillance Network
EPI_ISL_13498265	National Institute for Communicable Diseases of the National Health Laboratory Service	National Institute for Communicable Diseases of the National Health Laboratory Service	Chan WY, Mtshali PS, Grobbelaar A, Moolla N, Mohale T, Du Plessis MG, Ismail A, Weyer J
EPI_ISL_13502582	Laboratory of Microbiology and Virology, Ospedale Amedeo di Savoia, ASL "Città di Torino"	Laboratory of Microbiology and Virology, Ospedale Amedeo di Savoia, ASL "Città di Torino"	Francesco Cerutti, Antonella Bottoni, Marisa Cazzadore, Tiziano Allice, Maria Grazia Milia, Gabriella Gregori, Elisa Burdino, Valeria Ghisetti
EPI_ISL_13508393	Hosp. Itacolomy Butanta	Instituto Adolfo Lutz Strategic Laboratory	Claudio Tavares Sacchi, Karoline Rodrigues Campos, Ariadne Ferreira Amarante, Adriano Abbud, Adriana Bugno
EPI_ISL_13508471	Instituto de Infectologia Emilio Ribas	Instituto Adolfo Lutz Strategic Laboratory	Claudio Tavares Sacchi, Karoline Rodrigues Campos, Ariadne Ferreira Amarante, Adriano Abbud, Adriana Bugno
EPI_ISL_13511312	Laboratorio de Salud Pública de Antioquia	Instituto Nacional de Salud- Dirección de Investigación en Salud Pública	Katherine Laiton-Donato, Diego A. Álvarez-Díaz, Carlos Franco-Muñoz, Héctor A. Ruiz-Moreno, Paola Rojas-Estevez, Andres Prada, Alicia Rosales, Marcela Mercado-Reyes
EPI_ISL_13530881	Laboratorio de Referencia Nacional de Virus Respiratorios. Centro Nacional de Salud Publica. Instituto Nacional de Salud Peru.	Laboratorio de Referencia Nacional de Virus Respiratorios. Centro Nacional de Salud Publica. Instituto Nacional de Salud Peru.	Carlos Padilla Rojas, Veronica Hurtado Vela, Iris Silva Molina, Luren Sevilla Castañeda, Victor Jimenez Vasquez, Orson Mestanza Millones, Luis Barcena Flores, Wendy Lizarraga Olivares, Alicia Nuñez Llanos, Steve Acedo Lazo, Francisco Ascue Orosco, Kelly Izarra Rojas, Princesa Medrano Alhuay, Karla Vasquez Cajachahua, Estela Huaman Angeles, Jorge Giraldo Chavez, Lilian Huarca Balbin, Lisbet Roxana Inga Angulo, Maria Sandra Villar Saavedra, Henri Bailon Calderon, Lely Solari Zerpa, Gloria Arotinco Garayar. Equipo de vigilancia genomica del Instituto Nacional de Salud.
EPI_ISL_13537922	Instituto de Medicina Tropical de Sao Paulo (IMT-USP)	School of Public Health, Imperial College London	Coletti,T.M., Ghilardi,F., khan,M.J., Claro,I.M., Valenca,I.N., Faria,N.R. and Sabino,E.C.

EPI_ISL_13537923	Microbiology, Immunology and Transplantation, KU Leuven, Rega Institute	Microbiology, Immunology and Transplantation, KU Leuven, Rega Institute	Wawina-Bokalanga,T., Vanmechelen,B., Logist,A.-S., Sinnesael,R., Ysebaert,L., Bloemen,M. and Maes,P.
EPI_ISL_13537924, EPI_ISL_13537925, EPI_ISL_13537926	Microbiology, Immunology and Transplantation, KU Leuven, Rega Institute	Microbiology, Immunology and Transplantation, KU Leuven, Rega Institute	Vanmechelen,B., Wawina-Bokalanga,T., Logist,A.-S., Sinnesael,R., Ysebaert,L., Verlinden,J., Van Holm,B., Bloemen,M. and Maes,P.
EPI_ISL_13544223, EPI_ISL_13544224, EPI_ISL_13544225, EPI_ISL_13544226, EPI_ISL_13544227, EPI_ISL_13544228, EPI_ISL_13544229, EPI_ISL_13544230, EPI_ISL_13544231, EPI_ISL_13544232, EPI_ISL_13544233, EPI_ISL_13544234, EPI_ISL_13544235			
see above	Public Health Agency of Canada, National Microbiology Laboratory	Public Health Agency of Canada, National Microbiology Laboratory	Duggan,A., Hole,D., Knox,N., Yadav,C., Haidl,E., Chapel,M., Domselaar,G.V., Jolly,G., Audet,J., Fernando,L., Antonation,K., Safronetz,D., Hagan,M., Griffiths,E., Leung,A., Graham,M., Peters,G., Go,A., Laminman,V., Kaplen,B., Eshaghi,A., Gubbay,J.B., Hasso,M., Marchand-Austin,A., Olsha,R. and Patel,S.N.
EPI_ISL_13544239, EPI_ISL_13544240, EPI_ISL_13544241, EPI_ISL_13544243, EPI_ISL_13544244, EPI_ISL_13544245, EPI_ISL_13544246, EPI_ISL_13544247, EPI_ISL_13544248, EPI_ISL_13544249, EPI_ISL_13544250, EPI_ISL_13544251, EPI_ISL_13544252, EPI_ISL_13544253, EPI_ISL_13544254, EPI_ISL_13544255, EPI_ISL_13544256, EPI_ISL_13544257, EPI_ISL_13544258, EPI_ISL_13544259, EPI_ISL_13544260, EPI_ISL_13544261, EPI_ISL_13544262, EPI_ISL_13544263, EPI_ISL_13544264, EPI_ISL_13544265, EPI_ISL_13544266, EPI_ISL_13544267			
see above	Public Health Agency of Canada, National Microbiology Laboratory	Public Health Agency of Canada, National Microbiology Laboratory	Duggan,A., Hole,D., Knox,N., Yadav,C., Haidl,E., Chapel,M., Domselaar,G.V., Fernando,L., Graham,M., Antonation,K., Audet,J., Hagan,M., Safronetz,D., Leung,A., Peters,G., Go,A., Laminman,V., Kaplen,B., Jolly,G., Charest,H., Levade,I. and Fafard,J.
EPI_ISL_13573943	Center for Virology, Medical University of Vienna	Medical University of Vienna Center for Virology	Jeremy V. Camp, Monika Redlberger-Fritz, Stephan W. Aberle
EPI_ISL_13584854, EPI_ISL_13586184	Institute for Virology, Philipps-University Marburg	Institute for Virology, Philipps-University Marburg	Eickmann, M., Lier, C., Kowalski, K., Kraft, F., Becker, S.
EPI_ISL_13624509	Instituto de Diagnóstico y Referencia Epidemiológicos/Jurisdicción Sanitaria Cuauhtémoc/Hospital Ángeles Roma	Instituto de Diagnóstico y Referencia Epidemiológicos/Instituto de Biotecnología UNAM	Adnan Araiza-Rodríguez, Adriana Salvador-Patiño, Alejandro Sánchez-Flores, América del Pilar Mandujano-Martínez, Blanca Taboada, Carlos Eduardo Hernández-Sánchez, Carlos F. Arias, Claudia Elena Wong-Arámbula, Daniel José Regalado-Santiago, David Esaú Fragoso-Fonseca, Elizabeth Andrade-Montiel, Fabiola Garcés-Ayala, Fernando González-Dominguez, Gabriel García-Rodríguez, Gloria Vázquez-Castro, Hugo López Gatell Ramírez, Irma López-Martínez, Jerome Verleyen, Jesús Trujillo, Jorge Ochoa, José Ernesto Ramírez-González, Karel Estrada-Guerra, Lucía Hernández-Rivas, Magaly Guadalupe Landa-Flores, Maribel González-Villa, Mireya Mederos-Michel, Nancy Martínez-Velázquez, Noé Escobar-Escamilla, Oliva López, Ricardo Cortés-Alcalá, Ricardo Grande, Verónica Jiménez-Jacinto
EPI_ISL_13632071	Center of Diagnostics and Vaccine Development, Centers for Disease Control, Taiwan	Center of Diagnostics and Vaccine Development, Centers for Disease Control, Taiwan	Jih-Hui Lin, Shu-Chun Chiu, Hsin-I, Huang, Wei-Lun Huang, Wen-Bin, Fann, Pei-Yu, Hsieh, Jyh-Yuan Yang
EPI_ISL_13632288	National Institute for Communicable Diseases of the National Health Laboratory Service	National Institute for Communicable Diseases of the National Health Laboratory Service	Chan WY, Mtshali PS, Grobbelaar A, Moolla N, Mohale T, Lowe M, Du Plessis MG, Ismail A, Weyer J
EPI_ISL_13651348, EPI_ISL_13651349, EPI_ISL_13651350	Laboratorio de Referencia Nacional de Virus Respiratorio. Centro Nacional de Salud Publica. Instituto Nacional de Salud.	Laboratorio de Referencia Nacional de Virus Respiratorio. Centro Nacional de Salud Publica. Instituto Nacional de Salud.	Carlos Padilla Rojas, Veronica Hurtado Vela, Iris Silva Molina, Luren Sevilla Castañeda, Victor Jimenez Vasquez, Orson Mestanza Millones, Luis Barcena Flores, Wendy Lizarraga Olivares, Alicia Nuñez Llanos, Steve Acedo Lazo, Francisco Ascue Orosco, Kelly Izarra Rojas, Princesa Medrano Alhuay, Karla Vasquez Cajachahua, Estela Huaman Angeles, Jorge Giraldo Chavez, Lilian Huarca Balbin, Lisbet Roxana Inga Angulo, Maria Sandra Villar Saavedra, Henri Bailon Calderon, Lely Solari Zerpa, Gloria Arotinco Garayar. Equipo de vigilancia genomica del Instituto Nacional de Salud.
EPI_ISL_13658019, EPI_ISL_13658021	Erasmus Medical Center Department of Virology	Erasmus Medical Center Department of Virology	Bas Oude Munnink, Marjan Boter, Babette Weller, Richard Molenkamp, Janette Rahamat-Langendoen, Reina Sikkema, Marion Koopmans
EPI_ISL_13660191	Hospital Center Luxembourg	Laboratoire National de Santé Microbiology	Eric Hugoson, Ines Kozar, Sibel Berger, Anke Wienecke-Baldacchino, Bas Oude Munnink, Michel Kohnen, Jean-Hugues Francois, Tamir Abdelrahman
EPI_ISL_13705358	Hosp. Alemao Oswaldo Cruz	Instituto Adolfo Lutz Strategic Laboratory	Claudio Tavares Sacchi, Karoline Rodrigues Campos, Ariadne Ferreira Amarante, Marlon Benedito Nascimento Santos, Alex Domingos Reis, Adriano Abbud, Adriana Bugno
EPI_ISL_13705407	Hosp. Sirio-Libanes	Instituto Adolfo Lutz Strategic Laboratory	Claudio Tavares Sacchi, Karoline Rodrigues Campos, Ariadne Ferreira Amarante, Marlon Benedito Nascimento Santos, Alex Domingos Reis, Adriano Abbud, Adriana Bugno
EPI_ISL_13717674	Hospital Center Luxembourg	Laboratoire National de Santé Microbiology	Eric Hugoson, Ines Kozar, Sibel Berger, Anke Wienecke-Baldacchino, Bas Oude Munnink, Michel Kohnen, Jean-Hugues Francois, Tamir Abdelrahman
EPI_ISL_13728303	Department of Medical Microbiology & Infection prevention, Amsterdam University Medical Centers location AMC	Department of Medical Microbiology & Infection prevention, Amsterdam University Medical Centers location AMC	Matthijs Welkers, Jelle Koopsen, Robin van Houdt, Marcel Jonges, Sebastien Matamoros, Sjoerd Rebers, Fokla Zorgdrager, Sylvia Bruisten, Judith den Uil, Akke Cornelissen, Janke Schinkel, Menno de Jong, Gini van Rijckevorsel and Mariken van der Lubben on behalf of the Amsterdam Regional Genomic epidemiology and Outbreak Surveillance (ARGOS) consortium
EPI_ISL_13732932	Hosp. Sao Joaquim - Beneficiencia Portuguesa	Instituto Adolfo Lutz Strategic Laboratory	Claudio Tavares Sacchi, Karoline Rodrigues Campos, Ariadne Ferreira Amarante, Marlon Benedito Nascimento Santos, Alex Domingos Reis, Adriano Abbud, Adriana Bugno
EPI_ISL_13734233	Microbial Genomics, Hospital General Universitario Gregorio Maranon	Microbial Genomics, Hospital General Universitario Gregorio Maranon	Palomino-Cabrera,R., Penas-Utrilla,D., Buenestado-Serrano,S., Perez-Lago,L., Herranz Martin,M., Veintimilla,C., Catalan,P., Munoz,P. and Garcia de Viedma,D.
EPI_ISL_13734237, EPI_ISL_13734238, EPI_ISL_13734239, EPI_ISL_13734240, EPI_ISL_13734241, EPI_ISL_13734242, EPI_ISL_13734243, EPI_ISL_13734244, EPI_ISL_13734245, EPI_ISL_13734246, EPI_ISL_13734247, EPI_ISL_13734248, EPI_ISL_13734249, EPI_ISL_13734250, EPI_ISL_13734251, EPI_ISL_13734252, EPI_ISL_13734253, EPI_ISL_13734254, EPI_ISL_13734255, EPI_ISL_13734256, EPI_ISL_13734257, EPI_ISL_13734258, EPI_ISL_13734259, EPI_ISL_13734260, EPI_ISL_13734261, EPI_ISL_13734262, EPI_ISL_13734263, EPI_ISL_13734264, EPI_ISL_13734265, EPI_ISL_13734266, EPI_ISL_13734267, EPI_ISL_13734268			
see above	Centre for Biological Threats, Highly Pathogenic Viruses, Robert Koch Institute	Centre for Biological Threats, Highly Pathogenic Viruses, Robert Koch Institute	Brinkmann,A., Kohl,C., Pape,K., Uddin,S., Schrick,L., Michel,J., Schaade,L. and Nitsche,A.
EPI_ISL_13734269	Department of Clinical Sciences, Institute of Tropica Medicine	Department of Clinical Sciences, Institute of Tropica Medicine	De Baetselier,I., Van Dijck,C., Kenyon,C., Coppens,J., Smet,H., de Block,T., Coppens,S., Vanroye,F., Bugert,J., Girl,P., Liesenborghs,L., Selhorst,P., Arien,K., Van den Bossche,D., Florence,E., Rezende,A.M., Vercauteren,K. and Van Esbroeck,M.
EPI_ISL_13734270	Division of High-Consequence Pathogens and Pathology, Centers for Disease Control and Prevention	Division of High-Consequence Pathogens and Pathology, Centers for Disease Control and Prevention	Gigante,C.M., Ventura,J., Seabolt,M.H., Zhao,H., Wilkins,K., Respress,J., Howard,D., Batra,D., McCollum,A., Hutson,C., Davidson,W., Rao,A., Nash,J. and Li,Y.
EPI_ISL_13744896	Division of High-Consequence Pathogens and Pathology, Centers for Disease Control and Prevention	Division of High-Consequence Pathogens and Pathology, Centers for Disease Control and Prevention	Gigante,C.M., Ghinai,I., Seabolt,M.H., Zhao,H., Wilkins,K., Respress,J., Howard,D., Batra,D., McCollum,A., Hutson,C., Davidson,W., Rao,A., Kerins,J. and Li,Y.
EPI_ISL_13744897, EPI_ISL_13744898	Division of High-Consequence Pathogens and Pathology, Centers for Disease Control and Prevention	Division of High-Consequence Pathogens and Pathology, Centers for Disease Control and Prevention	Gigante,C.M., Hughes,S., Seabolt,M.H., Zhao,H., Wilkins,K., Respress,J., Howard,D., Batra,D., McCollum,A., Hutson,C., Davidson,W., Rao,A., Baumgartner,J. and Li,Y.
EPI_ISL_13744899	Division of High-Consequence Pathogens and Pathology, Centers for Disease Control and Prevention	Division of High-Consequence Pathogens and Pathology, Centers for Disease Control and Prevention	Gigante,C.M., Ghinai,I., Seabolt,M.H., Zhao,H., Wilkins,K., Respress,J., Howard,D., Batra,D., McCollum,A., Hutson,C., Davidson,W., Rao,A., Kerins,J. and Li,Y.
EPI_ISL_13744900, EPI_ISL_13744901	Division of High-Consequence Pathogens and Pathology, Centers for Disease Control and Prevention	Division of High-Consequence Pathogens and Pathology, Centers for Disease Control and Prevention	Gigante,C.M., Hughes,S., Seabolt,M.H., Zhao,H., Wilkins,K., Respress,J., Howard,D., Batra,D., McCollum,A., Hutson,C., Davidson,W., Rao,A., Baumgartner,J. and Li,Y.
EPI_ISL_13744902	Department of Virology, Faculty of Medicine, University of Helsinki	Department of Virology, Faculty of Medicine, University of Helsinki	Kant,R., Smura,T., Vauhkonen,H. and Vapalahti,O.
EPI_ISL_13744903, EPI_ISL_13744904, EPI_ISL_13744905	Centre for Biological Threats, Highly Pathogenic Viruses, Robert Koch Institute	Centre for Biological Threats, Highly Pathogenic Viruses, Robert Koch Institute	Brinkmann,A., Kohl,C., Pape,K., Uddin,S., Schrick,L., Michel,J., Jessen,H., Schaade,L. and Nitsche,A.
EPI_ISL_13744906, EPI_ISL_13744907, EPI_ISL_13744908, EPI_ISL_13744909, EPI_ISL_13744910, EPI_ISL_13744911, EPI_ISL_13744912, EPI_ISL_13744913, EPI_ISL_13744914, EPI_ISL_13744915, EPI_ISL_13744916, EPI_ISL_13744917, EPI_ISL_13744918, EPI_ISL_13744919, EPI_ISL_13744920, EPI_ISL_13744921, EPI_ISL_13744922, EPI_ISL_13744923, EPI_ISL_13744924, EPI_ISL_13744925, EPI_ISL_13744926, EPI_ISL_13744927, EPI_ISL_13744928, EPI_ISL_13744929, EPI_ISL_13744930, EPI_ISL_13744931			
see above	Centre for Biological Threats, Highly Pathogenic Viruses, Robert Koch Institute	Centre for Biological Threats, Highly Pathogenic Viruses, Robert Koch Institute	Brinkmann,A., Kohl,C., Pape,K., Uddin,S., Schrick,L., Michel,J., Schaade,L. and Nitsche,A.
EPI_ISL_13822667, EPI_ISL_13822668, EPI_ISL_13822669, EPI_ISL_13822718	Erasmus Medical Center Department of Virology	Erasmus Medical Center Department of Virology	Bas Oude Munnink, Marjan Boter, Babette Weller, Richard Molenkamp, Janette Rahamat-Langendoen, Reina Sikkema, Marion Koopmans
EPI_ISL_13827273, EPI_ISL_13827274, EPI_ISL_13827275, EPI_ISL_13827277, EPI_ISL_13827278, EPI_ISL_13827279,	Public Health Agency of Canada, National Microbiology Laboratory	Public Health Agency of Canada, National Microbiology Laboratory	Duggan,A., Hole,D., Yadav,C., Knox,N., Haidl,E., Chapel,M., Domselaar,G.V., Fernando,L., Graham,M., Antonation,K., Audet,J., Hagan,M., Safronetz,D., Leung,A., Peters,G., Go,A., Laminman,V., Kaplen,B., Jolly,G., Marchand-Austin,A., Eshaghi,A., Patel,S.N., Hasso,M., Gubbay,J.B. and Olsha,R.

EPI_ISL_13827280, EPI_ISL_13827281, EPI_ISL_13827282			
EPI_ISL_13833194, EPI_ISL_13833195, EPI_ISL_13833196, EPI_ISL_13833197	Laboratorio de Referencia Nacional de Virus Respiratorio. Centro Nacional de Salud Publica. Instituto Nacional de Salud.	Laboratorio de Referencia Nacional de Virus Respiratorio. Centro Nacional de Salud Publica. Instituto Nacional de Salud.	Carlos Padilla Rojas, Veronica Hurtado Vela, Iris Silva Molina, Luren Sevilla Castañeda, Victor Jimenez Vasquez, Orson Mestanza Millones, Luis Barcena Flores, Wendy Lizarraga Olivares, Alicia Nuñez Llanos, Steve Acedo Lazo, Francisco Ascue Orosco, Kelly Izarra Rojas, Princesa Medrano Alhuay, Karla Vasquez Cajachahua, Estela Huaman Angeles, Jorge Giraldo Chavez, Lilian Huarca Balbin, Lisbet Roxana Inga Angulo, Maria Sandra Villar Saavedra, Henri Bailon Calderon, Lely Solari Zerpa, Gloria Arotinco Garayar. Equipo de vigilancia genomica del Instituto Nacional de Salud.
EPI_ISL_13842269, EPI_ISL_13842548	Center for Virology, Medical University of Vienna	Medical University of Vienna Center for Virology	Jeremy V. Camp, Monika Redlberger-Fritz, Stephan W. Aberle
EPI_ISL_13889435, EPI_ISL_13889436, EPI_ISL_13889438, EPI_ISL_13889439, EPI_ISL_13889440, EPI_ISL_13889441, EPI_ISL_13889442, EPI_ISL_13889443, EPI_ISL_13889444, EPI_ISL_13889445, EPI_ISL_13889446, EPI_ISL_13889447, EPI_ISL_13889448, EPI_ISL_13889449, EPI_ISL_13889450, EPI_ISL_13889515, EPI_ISL_13889590, EPI_ISL_13889660, EPI_ISL_13889729, EPI_ISL_13889796, EPI_ISL_13889908, EPI_ISL_13889977, EPI_ISL_13890048, EPI_ISL_13890135, EPI_ISL_13890204, EPI_ISL_13890273, EPI_ISL_13890338, EPI_ISL_13890408, EPI_ISL_13890464, EPI_ISL_13890465, EPI_ISL_13890466, EPI_ISL_13890467, EPI_ISL_13890468, EPI_ISL_13890469, EPI_ISL_13890470, EPI_ISL_13890471, EPI_ISL_13890472, EPI_ISL_13890473, EPI_ISL_13890474, EPI_ISL_13890475, EPI_ISL_13890476, EPI_ISL_13890477, EPI_ISL_13890478, EPI_ISL_13890479, EPI_ISL_13890481, EPI_ISL_13890482			
see above	Charité Universitätsmedizin Berlin, Institut für Virologie/Labor Berlin	Charité Universitätsmedizin Berlin, Institut für Virologie	Terry C. Jones, Julia Schneider, Barbara Mühlemann, Talitha Veith, Jörn Beheim-Schwarzbach, Julia Tesch, Marie Luisa Schmidt, Felix Walper, Tobias Bleicker, Caroline Isner, Frieder Pfäfflin, Ricardo Niklas Werner, Victor M. Corman, Christian Drosten
EPI_ISL_13891126	Ministry of Health Turkey	Ministry of Health Turkey	Fatma Bayrakdar, Suleyman Yalcin, Gulay Korukluoglu
EPI_ISL_13908328	Center of Diagnostics and Vaccine Development, Centers for Disease Control	Center of Diagnostics and Vaccine Development, Centers for Disease Control	Lin,J.-H., Chiu,S.-C., Huang,H.-I., Huang,W.-L., Fann,W.-B., Hsieh,P.-Y., Hsu,S.-C., Liu,P.-C., Chang,T.-Y. and Yang,J.-Y.
EPI_ISL_13908329, EPI_ISL_13908332, EPI_ISL_13908333, EPI_ISL_13908334, EPI_ISL_13908335, EPI_ISL_13908336, EPI_ISL_13908337, EPI_ISL_13908338, EPI_ISL_13908339, EPI_ISL_13908340, EPI_ISL_13908341, EPI_ISL_13908342, EPI_ISL_13908343, EPI_ISL_13908345			
see above	Public Health Agency of Canada, National Microbiology Laboratory	Public Health Agency of Canada, National Microbiology Laboratory	Duggan,A., Hole,D., Yadav,C., Knox,N., Chapel,M., Tyler,A., Haidl,E., Domselaar,G.V., Antonation,K., Audet,J., Fernando,L., Hagan,M., Safronetz,D., Graham,M., Peters,G., Go,A., Laminman,V., Kaplen,B., Leung,A., Jolly,G., Fafard,J., Charest,H. and Levade,I.
EPI_ISL_13908346, EPI_ISL_13908347, EPI_ISL_13908348, EPI_ISL_13908349	Centre for Biological Threats, Highly Pathogenic Viruses, Robert Koch Institute	Centre for Biological Threats, Highly Pathogenic Viruses, Robert Koch Institute	Brinkmann,A., Kohl,C., Pape,K., Uddin,S., Schrick,L., Michel,J., Jessen,H., Schaade,L. and Nitsche,A.
EPI_ISL_13908350, EPI_ISL_13908351, EPI_ISL_13908352, EPI_ISL_13908353, EPI_ISL_13908354, EPI_ISL_13908355, EPI_ISL_13908356, EPI_ISL_13908357, EPI_ISL_13908358, EPI_ISL_13908359, EPI_ISL_13908360, EPI_ISL_13908361, EPI_ISL_13908362, EPI_ISL_13908363, EPI_ISL_13908364, EPI_ISL_13908365			
see above	Centre for Biological Threats, Highly Pathogenic Viruses, Robert Koch Institute	Centre for Biological Threats, Highly Pathogenic Viruses, Robert Koch Institute	Brinkmann,A., Kohl,C., Pape,K., Uddin,S., Schrick,L., Michel,J., Schaade,L. and Nitsche,A.
EPI_ISL_13953610, EPI_ISL_13953611	Indian Council of Medical Research-National Institute of Virology	Indian Council of Medical Research-National Institute of Virology	Pragya Yadav, Rima Sahay, Anita Aich Shete, Sreelekshmy Mohandas, Priya Abraham
EPI_ISL_13955501	Public Health Authority of the Slovak Republic	Laboratory of Genomics and Bioinformatics, Comenius University Science Park	Tomáš Szemes, Edita Staroová, Elena Tichá, Lucia Ševíková, Terézia Vrabová, Tatiana Sedláková, Miroslav Böhmer, Jaroslav Budiš, Pavol Mišenko
EPI_ISL_13958697	Research and Evaluation, UKHSA	Research and Evaluation, UKHSA	Groves,N., Osman,K.L., Lewandowski,K.S., Carter,D.P., Pullan,S.T., Myers,R., Vipond,R. and Chand,M.
EPI_ISL_13983354, EPI_ISL_13983355	Instituto de Infectologia Emilio Ribas	Instituto Adolfo Lutz Strategic Laboratory	Claudio Tavares Sacchi, Karoline Rodrigues Campos, Ariadne Ferreira Amarante, Marlon Benedito Nascimento Santos, Alex Domingos Reis, Adriano Abbud, Adriana Bugno
EPI_ISL_13983356	INSPI-Centro de Referencia Nacional de Virus Exantemáticos, Gastroentéricos y Transmitido por Vectores.	INSPI-Dirección Técnica de Investigación, Desarrollo e Innovación INSPI-Centro de Referencia Nacional de Genómica, Secuenciación y Bioinformática	Andrés Carrazco-Montalvo, Diana Gutiérrez, Naomi Mora, Silvia Salgado-Cisneros, Johana Parrales-Valdiviezo, Martha Sánchez-Domenech, Diego Morales, Gulnara Borja-Cabrera, Leandro Patiño*.
EPI_ISL_13983888	Bangkok Hospital Phuket	National Institute of Health, Department of Medical Sciences, Ministry of Public Health, Thailand	Pilailuk Okada; Siripaporn Phuygun; Nuttida Thongpramul; Thanutsapa Thanadachakul; Kazuhisa Okada; Archawin Rojanawiwat; Chakkarat Pitayawonganon; Supakit Sirilak
EPI_ISL_13993734, EPI_ISL_13993735, EPI_ISL_13993736, EPI_ISL_13993737, EPI_ISL_13993738, EPI_ISL_13993739	California Department of Public Health	California Department of Public Health	Viral and Rickettsial Disease Laboratory
EPI_ISL_14003930	URMC LABS	URMC LABS	Andrew Cameron, Mondraya Howard, Sara Connelly, Dwight Hardy, Kelly DeLary
EPI_ISL_14011193	Bangkok Hospital Phuket	Thai Red Cross Emerging Infectious Diseases Clinical Center and Faculty of Medicine, Chulalongkorn University	Kusak Kukiattikoon, Waritta Dararattanaroj, Rome Buathong, Supaporn Wacharapluesadee, Sininat Petcharat, Ananporn Supataragul, Stefan Fernandez, Achawin Rojanaviwat, Chonticha Klungthong, Pilailuk Okada, Khajohn Joonlasak, Chakkarat Pitayawonganon, Opass Putcharoen
EPI_ISL_14021725	Hosp. Municipal Enf. Antonio Policarpo de Oliveira	Instituto Adolfo Lutz Strategic Laboratory	Claudio Tavares Sacchi, Karoline Rodrigues Campos, Ariadne Ferreira Amarante, Marlon Benedito Nascimento Santos, Alex Domingos Reis, Adriano Abbud, Adriana Bugno
EPI_ISL_14033204, EPI_ISL_14033205, EPI_ISL_14033206, EPI_ISL_14033207, EPI_ISL_14033208, EPI_ISL_14033209, EPI_ISL_14033210, EPI_ISL_14033211, EPI_ISL_14033212, EPI_ISL_14033213	Laboratory Medicine, UW Virology	Laboratory Medicine, UW Virology	Sereewit,J., Xie,H., Pavitra,R. and Greninger,A.
EPI_ISL_14049244, EPI_ISL_14049245	Indian Council of Medical Research-National Institute of Virology	Indian Council of Medical Research-National Institute of Virology	Pragya Yadav, Rima Sahay, Anita Aich Shete, Sreelekshmy Mohandas, Priya Abraham
EPI_ISL_14050451, EPI_ISL_14050453, EPI_ISL_14050454, EPI_ISL_14050455, EPI_ISL_14050456, EPI_ISL_14050458	Public Health Agency of Canada, National Microbiology Laboratory	Public Health Agency of Canada, National Microbiology Laboratory	Duggan,A., Hole,D., Yadav,C., Knox,N., Tyler,A., Haidl,E., Chapel,M., Domselaar,G.V., Graham,M., Audet,J., Fernando,L., Hagan,M., Safronetz,D., Leung,A., Peters,G., Go,A., Laminman,V., Kaplen,B., Antonation,K., Jolly,G., Griffiths,E., Charest,H., Levade,I. and Fafard,J.
