# Supplementary material for: Vaccinia-Virus-Based Vaccines Are Expected to Elicit Highly Cross-Reactive Immunity to the 2022 Monkeypox Virus
Source: Viruses. 2022 Sep 3;14(9):1960. doi: 10.3390/v14091960 (PMC9506226; doi:10.3390/v14091960)
Supplement: Supplementary file 1 [file viruses-14-01960-s001.zip › Supplementary_Figs_S1_to_S3.pdf]

Vaccinia-virus-based vaccines are expected to elicit highly cross-reactive immunity to the 2022 monkeypox virus

Syed Faraz Ahmed [1,†], Muhammad Saqib Sohail [2,†], Ahmed Abdul Quadeer [2,*] and Matthew R. McKay [1,3,*]

[1] Department of Electrical and Electronic Engineering, University of Melbourne, Parkville, VIC 3010, Australia; faraz.ahmed@unimelb.edu.au (S.F.A.)
[2] Department of Electronic and Computer Engineering, The Hong Kong University of Science and Technology, Hong Kong SAR, China; mssohail@connect.ust.hk (M.S.S.)
[3] Department of Microbiology and Immunology, The Peter Doherty Institute for Infection and Immunity, University of Melbourne, Melbourne, VIC 3000, Australia
* Correspondence: eeaaquadeer@ust.hk (A.A.Q.); matthew.mckay@unimelb.edu.au (M.R.M.)
† These authors contributed equally to the work.

SUPPLEMENTARY FIGURES

SNP	VACV	MVA-BN	ACAM2000	Dryvax
VACV (194711 bp)		1%	2%	2%
MVA-BN (165041 bp)	2535		1%	1%
ACAM2000 (199234 bp)	3782	1309		<1%
Dryvax (198734 bp)	3723	1214	560	

Supplementary Figure S1. Full-genome comparison of the reference sequences for VACV and the three vaccines: MVA-BN, ACAM2000 and Dryvax (consensus sequence). Single nucleotide polymorphisms (SNPs) are indicated in the lower left triangle, while the percentage of SNPs are indicated on the upper right triangle. The percentage of SNPs are computed for the sequence indicated in each row where the length of the sequence is in parenthesis.

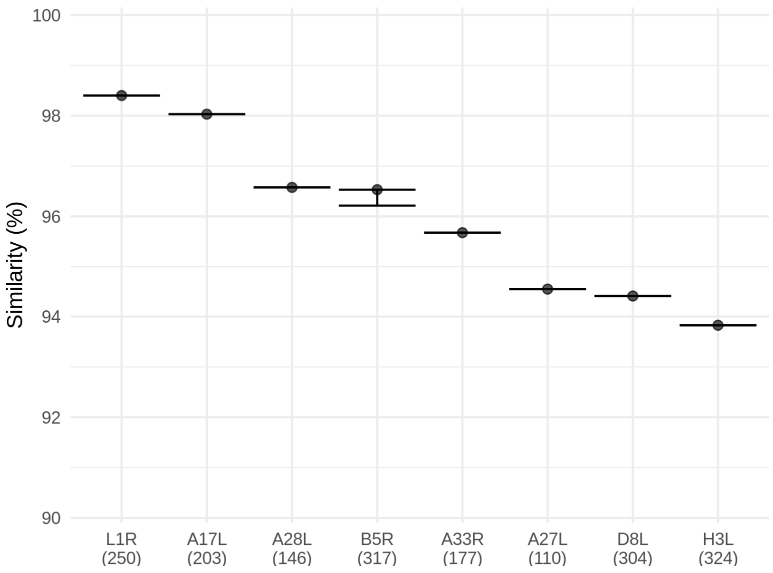

Supplementary Figure S2. Genetic similarity between VACV proteins targeted by NAbs and their orthologs in 531 MPXV-2022 sequences. A circle represents the median similarity of the VACV protein among the MPXV-2022 sequences, while the lower and upper bars indicate the 5th and 95th percentiles respectively. Length of the VACV proteins is indicated within parentheses on the x-axis.

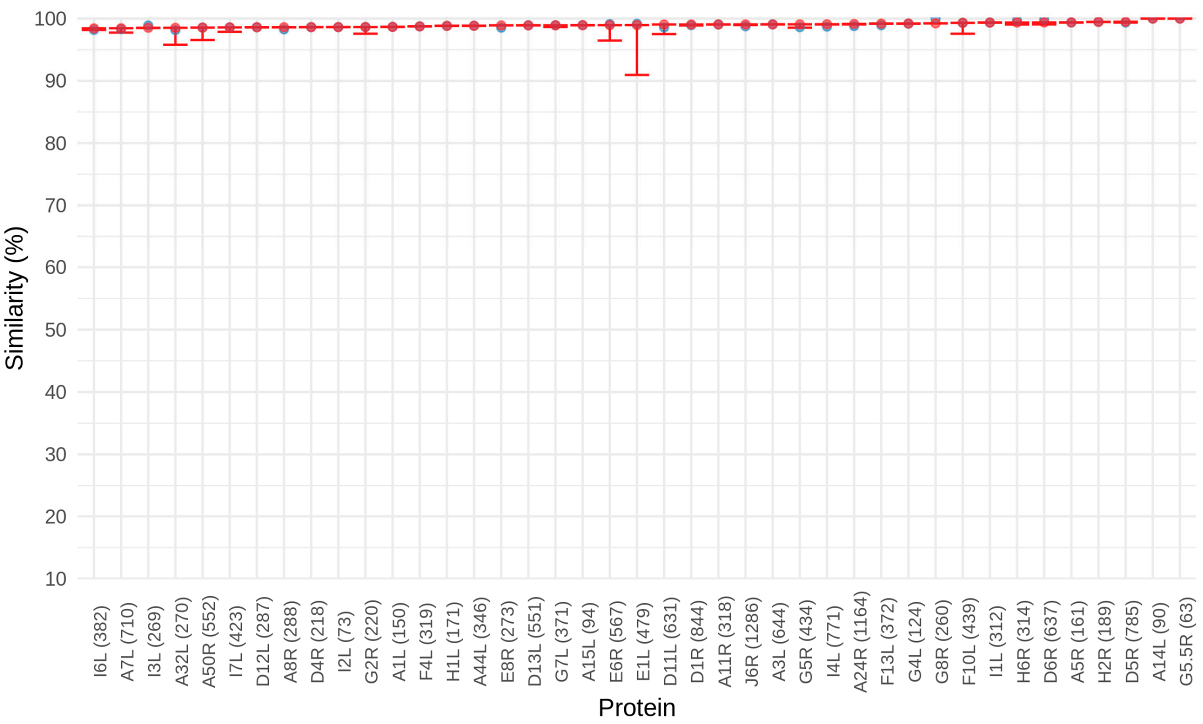
Similarity (%)
100
90
80
70
60
50
40
30
20
10
I6L (382)
A7L (710)
I3L (269)
A32L (270)
A50R (552)
I7L (423)
D12L (287)
A8R (288)
D4R (218)
I2L (73)
G2R (220)
A1L (150)
F4L (319)
H1L (171)
A44L (346)
E8R (273)
D13L (551)
G7L (371)
A15L (94)
E6R (567)
E1L (479)
D11L (631)
D1R (844)
A11R (318)
J6R (1286)
A3L (644)
G5R (434)
I4L (771)
A24R (1164)
F13L (372)
G4L (124)
G8R (260)
F10L (439)
I1L (312)
H6R (314)
D6R (637)
A5R (161)
H2R (189)
D5R (785)
A14L (90)
G5.5R (63)
Protein

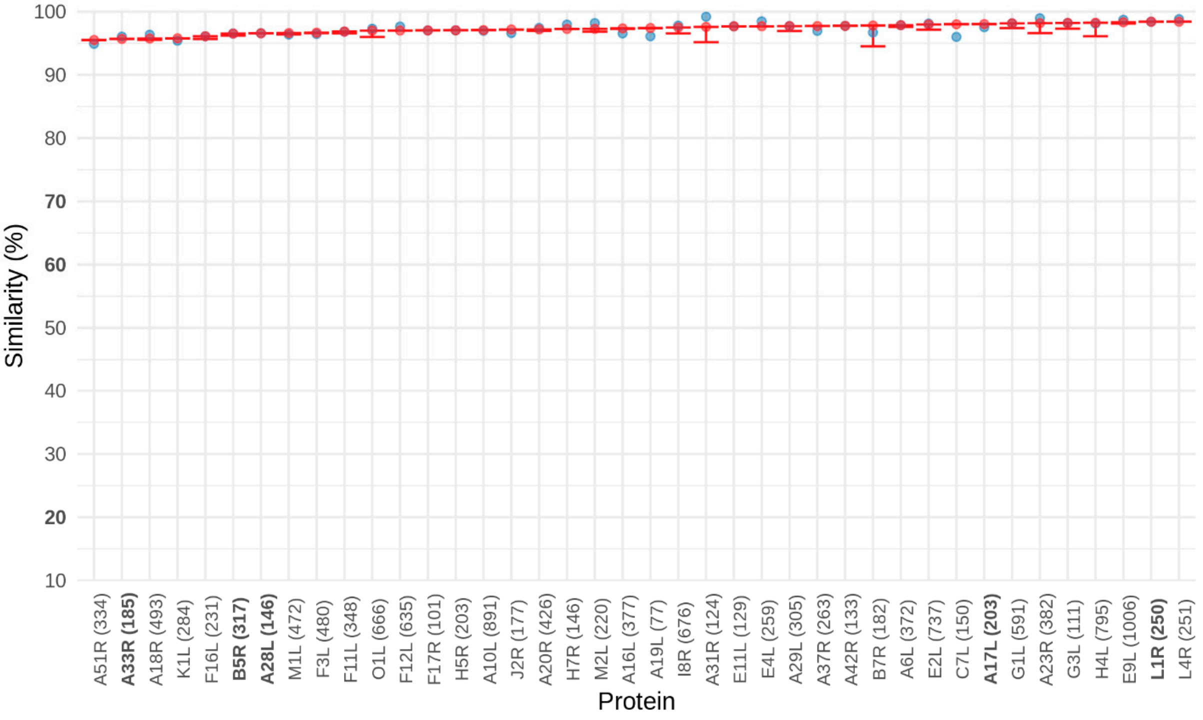
Similarity (%)
100
90
80
70
60
50
40
30
20
10
A51R (334)
A33R (185)
A18R (493)
K1L (284)
F16L (231)
B5R (317)
A28L (146)
M1L (472)
F3L (480)
F11L (348)
O1L (666)
F12L (635)
F17R (101)
H5R (203)
A10L (891)
J2R (177)
A20R (426)
H7R (146)
M2L (220)
A16L (377)
A19L (77)
I8R (676)
A31R (124)
E11L (129)
E4L (259)
A29L (305)
A37R (263)
A42R (133)
B7R (182)
A6L (372)
E2L (737)
C7L (150)
A17L (203)
G1L (591)
A23R (382)
G3L (111)
H4L (795)
E9L (1006)
L1R (250)
L4R (251)
Protein

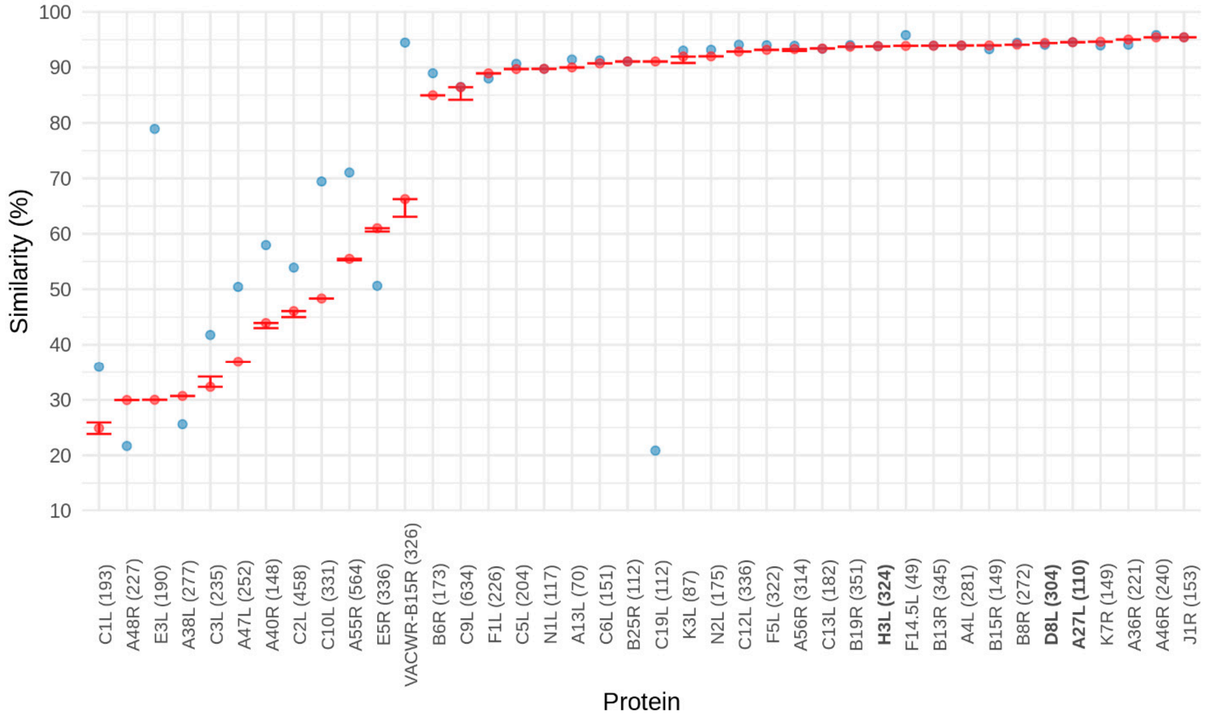

Supplementary Figure S3. Genetic similarity of 121 VACV-specific proteins with known T cell epitopes among MPXV-2022 (red) and MPXV-CB (blue) orthologs. Variation among the MPXV-2022 sequences is also shown where the lower and upper ends of the error bars indicate the 5th and 95th percentiles respectively. Note that for most proteins the diversity is limited and hence the two ends of the error bars overlap each other. VACV proteins known to be targets of NAbs are indicated in bold. For the twelve least similar VACV proteins (C1L, A48R, E3L, A38L, C3L, A47L, A40R, C2L, C10L, A55R, E5R and VACWR-B15R), there are large spans of indels, which may be a result of partial gene deletions and/or poor quality of the sequence alignment spanning genomic regions that encode these proteins.

SUPPLEMENTARY TABLES

Supplementary Table S1 (included as a separate file). List of GISAID Accession IDs of the MPXV-2022 sequences analyzed.

Supplementary Table S2 (included as a separate file). Complete list of experimentally-determined VACV T cell epitopes (n=388) and their conservation in MPXV-2022 and MPXV-CB.

Supplementary Table S3 (included as a separate file). Complete list of experimentally-determined VACV T cell epitopes (n=388) and their conservation in the reference sequences of MVA-BN, ACMA2000, and Dryvax vaccines.

Supplementary Table S4 (included as a separate file). Acknowledgment table (downloaded from GISAID).
